# PAK5 promotes the trastuzumab resistance by increasing HER2 nuclear accumulation in HER2-positive breast cancer

**DOI:** 10.1038/s41419-025-07657-2

**Published:** 2025-04-21

**Authors:** Xin Zhao, Yang Li, Hongyan Zhang, Yihang Cai, Xu Wang, Yidu Liu, Tingting Li, Chendong Xu, Yuee Teng, Danni Li, Feng Li

**Affiliations:** 1 Department of Cell Biology, Key Laboratory of Cell Biology, National Health Commission of the PRC and Key Laboratory of Medical Cell Biology, Ministry of Education of the PRC, China Medical University, Shenyang, Liaoning China; 2 Department of Pediatric Orthopaedics, Shengjing Hospital of China Medical University, Shenyang, Liaoning China; 3 Department of Breast Surgery, Department of Surgical Oncology, Research Unit of General Surgery, The First Affiliated Hospital of China Medical University, Shenyang, Liaoning China; 4 Department of Medical Oncology, The First Hospital of China Medical University, Shenyang, China

**Keywords:** Phosphorylation, Breast cancer

## Abstract

Nuclear HER2 (N-HER2) predicts resistance to HER2-targeted therapy and poor prognosis of breast cancer patients, and the underlying mechanisms remain unclear. Here, we show that high expression of p21-activated kinase 5 (PAK5) is associated with HER2-targeted therapy resistance and poor outcomes of breast cancer patients. Excitingly, we find an increase in N-HER2 protein expression in patients with high PAK5 expression, who demonstrate resistance to trastuzumab treatment. PAK5 phosphorylates methyltransferase METTL14 on serine 399 to enhance m^6^A modification of lncRNA metastasis-associated lung adenocarcinoma transcript 1 (MALAT1), leading to increased MALAT1 stability. The stabilized MALAT1 inhibits ubiquitin-proteasomal degradation of the N-HER2 by affecting the interaction of deubiquitinase USP8 and N-HER2, thereby promoting N-HER2 accumulation. Moreover, HER2 upregulates the expression of PAK5 and MALAT1, activating the HER2-MALAT1 positive feedback loop. Importantly, PAK5 promotes the therapeutic resistance of HER2-positive breast cancer cells by increasing N-HER2 protein both in vitro and vivo. These findings highlight PAK5 as a therapeutic target for combating trastuzumab resistance in HER2-positive breast cancer.

## Introduction

About 15–20% of breast cancer patients are HER2 positive, with characteristics of poor differentiation and strong invasion [1].

HER2 is a transmembrane tyrosine kinase receptor that belongs to the human epidermal growth factor (EGF) receptor family [2]. There has been notable progress in the development of HER2-targeting monoclonal antibodies [3–5], tyrosine kinase inhibitors (TKIs, lapatinib. etc), advanced HER2-targeting drugs like trastuzumab emtansine (T-DM1) and trastuzumab deruxtecan (T-DXd). Notwithstanding its established efficacy, drug resistance remains a major clinical problem, and most patients eventually progress deteriorating. Membrane HER2 (M-HER2) overexpression has a critical role in breast cancer. Current anti-HER2 therapies, as with the antibody trastuzumab, target only M-HER2. It has been reported that the presence of nuclear HER2 (N-HER2) in breast cancer, whose presence as a poor prognostic factor in HER2 positive tumors [6, 7]. N-HER2 is the major proliferation driver in trastuzumab-resistant breast cancer [8]. It is of great significance to elucidate the mechanism of trastuzumab and other HER2-targeting drugs resistance in HER2-positive breast cancer.

P21-activated kinase 5 (PAK5), a member of the PAK family of Ser/Thr kinases [9–13], phosphorylates a variety of proteins to promote breast cancer cell proliferation and metastasis. Besides, PAK5 regulates epithelial ovarian cancer cell paclitaxel chemoresistance [14]. PAK5-mediated phosphorylation and nuclear translocation of β-catenin facilitates ABCB1 regulation and confers the chemoresistant phenotype in hepatocellular carcinoma [15]. However, it is unknown whether PAK5 is involved in trastuzumab resistance in HER2-positive breast cancer.

Long non-coding RNA metastasis-associated lung adenocarcinoma transcript 1 (MALAT1) is a conserved long non-coding RNA [16–19]. Current studies show that MALAT1 is a potential target not only for cancer therapy, but also for overcoming cancer resistance [17]. Knockdown of MALAT1 in trastuzumab-resistant HER2 positive cells reversed epithelial to mesenchymal transition-like phenotype and cell invasiveness [20]. FOXO1 plays a regulatory role in mediating MALAT1 expression in breast cancer cells via the PI3K/Akt pathway [20]. Trastuzumab-resistant cells rely on HER2/PI3K/AKT/Survivin pathway to promote proliferation of cancer stem cells [21]. However, the connection of N-HER2 with MALAT1 in trastuzumab-resistant HER2-positive cells is not known.

N^6^-methyladenosine (m^6^A) modification, the most abundant post-transcriptional modification in eukaryotes, exerts important roles in regulating RNA metabolism [22–24]. Methyltransferase-like 14 (METTL14) is a key component to catalyze m^6^A modification on mRNA or non-coding RNA to regulate its expression and cell phenotypes [22, 25]. METTL14 is also involved in tumor chemotherapy [26]. However, the relationship between METTL14 and HER2-targeted therapy resistance in breast cancer is still unclear.

Ubiquitination and deubiquitination play key roles in reversible regulation of protein stability. USP8 reportedly deubiquitinates and stabilizes several substrate proteins such as HER2 [27] and ERα [28], indicating that USP8 is crucial in the development and progression of breast cancer and the underlying mechanisms remain unclear.

In this study, we observe an increase in N-HER2 protein expression in patients with high PAK5 expression, who exhibit resistance to trastuzumab treatment. PAK5 phosphorylates methyltransferase METTL14 to enhance m^6^A modification of lncRNA MALAT1, leading to increased MALAT1 stability. Moreover, MALAT1 inhibits ubiquitin-proteasomal degradation of the N-HER2 by affecting the binding of deubiquitinase USP8 and N-HER2, thereby promoting N-HER2 accumulation and trastuzumab resistance in HER2-positive breast cancer. These data identify PAK5 as a potential prognostic factor and a novel therapeutic target against trastuzumab resistance in breast cancer.

## Results

### A high level of PAK5 protein is associated with drug resistance in HER2-positive breast cancer patients receiving trastuzumab treatment

**Fig. 1:**
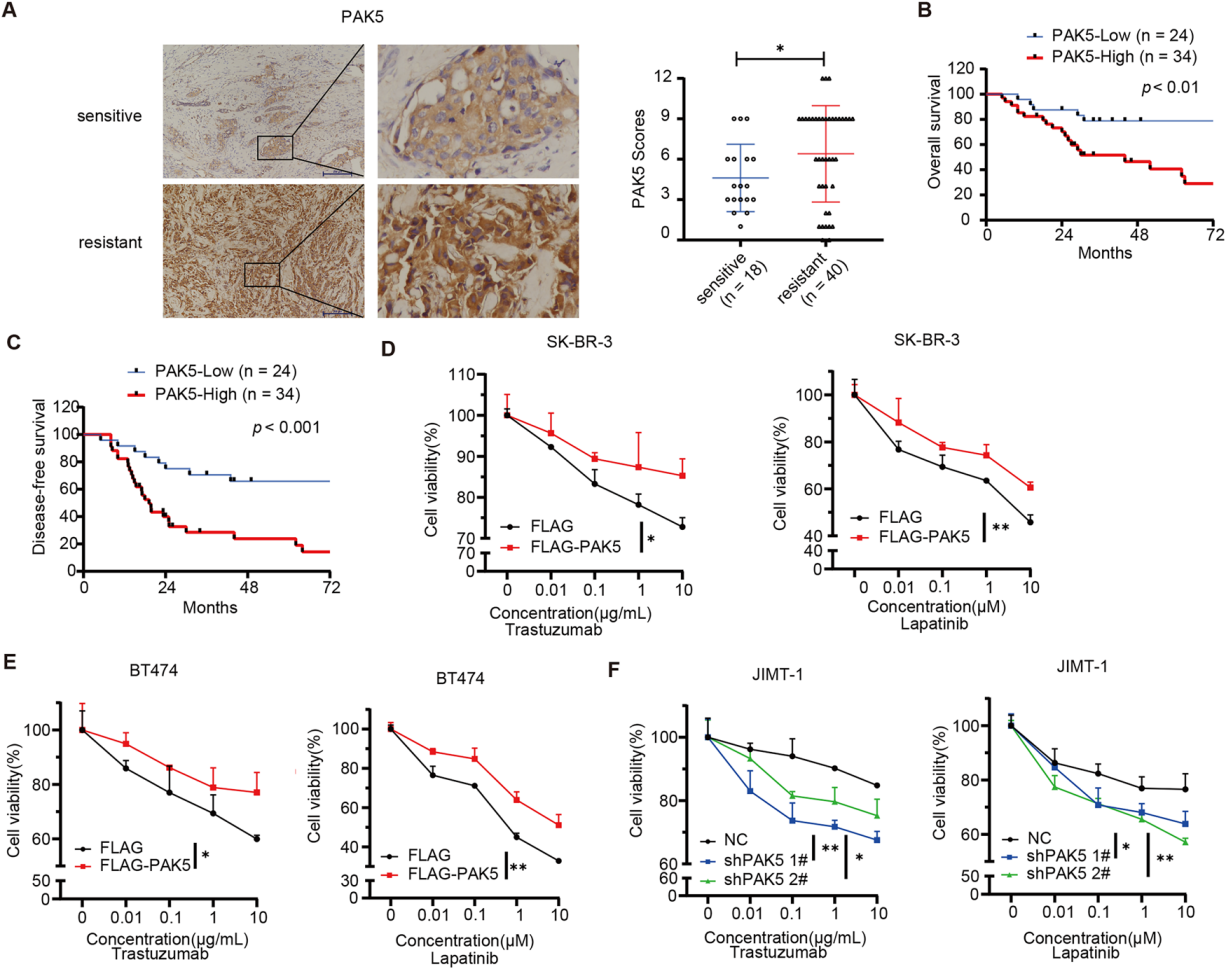
A high level of PAK5 protein is associated with drug resistance in HER2-positive breast cancer patients receiving trastuzumab treatment. **A** Representative images of PAK5 staining by IHC analysis in breast cancer specimens treated with trastuzumab (left). The boxed areas in the left images were magnified in the right images. Original magnification, ×100. IHC staining scores for PAK5 in HER2-positive breast cancer tissues from sensitive patients (*n* = 18) and resistant patients (*n* = 40) (right). *p < 0.05, *t*-test. **B** Overall survival (OS) curves of 58 breast cancer patients with low expression (*n* = 24) versus high expression of PAK5 (*n* = 34). **C** Disease-free survival (DFS) curves of 58 breast cancer patients with low expression (*n* = 24) versus high expression of PAK5 (*n* = 34). SK-BR-3 (**D**) and BT474 (**E**) cells stably expressing FLAG vector or FLAG -PAK5 were treated with trastuzumab or lapatinib at indicated concentrations for 72 h, cell viability was evaluated by CCK8 assay (*n* = 3 biological replicates). *p < 0.05, **p < 0.01, *t*-test. **F** JIMT-1 cells stably expressing PAK5 shRNA or control NC were treated with trastuzumab or lapatinib at indicated concentrations for 72 h, cell viability was evaluated by CCK8 assay (*n* = 3 biological replicates). *p < 0.05, **p < 0.01, one-way ANOVA.

To further investigate the molecular basis of PAK5 in breast cancer, we examined the PAK5 expression level in 102 breast cancer specimens using immunoblot analysis. Stratification analysis showed that the expression of PAK5 protein in breast cancer tissues was positively correlated with clinical N stage (*p* = 0.0177), metastasis (*p* = 0.0245), and HER2 status (*p* = 0.0321; Fig. S1A and Table S1). We also examined the expression of PAK5 in HER2 positive breast cancer cell lines (SK-BR-3, BT474, JIMT-1). Interestingly, a higher level of PAK5 protein was observed in the intrinsically trastuzumab-resistant JIMT-1 breast cancer cells compared to the trastuzumab-sensitive SK-BR-3 and BT474 cells (Fig. S1B). We verified the expression level of PAK5 by using IHC staining in HER2 positive breast cancer patients (*n* = 58) treated with trastuzumab, and found that patients with high expression of PAK5 were resistant to trastuzumab treatment (Fig. 1A). In addition, patients with high PAK5 expression breast cancer who received trastuzumab had poor overall survival (OS) and disease-free survival (DFS) (Fig. 1B, C). The CCK8 assays revealed that overexpression of PAK5 in the SK-BR-3 and BT474 cells reduced the sensitivity to trastuzumab- and lapatinib-mediated growth inhibition (Figs. 1D, E and S1C, D). In contrast, PAK5 knockdown significantly increased trastuzumab- and lapatinib-mediated growth inhibition in JIMT-1 cells (Figs. 1F and S1E). These data indicated that increased PAK5 expression may play an important role in the resistance of HER2-targeted therapies.

### PAK5 increases the N-HER2 protein level via lncRNA MALAT1

**Fig. 2:**
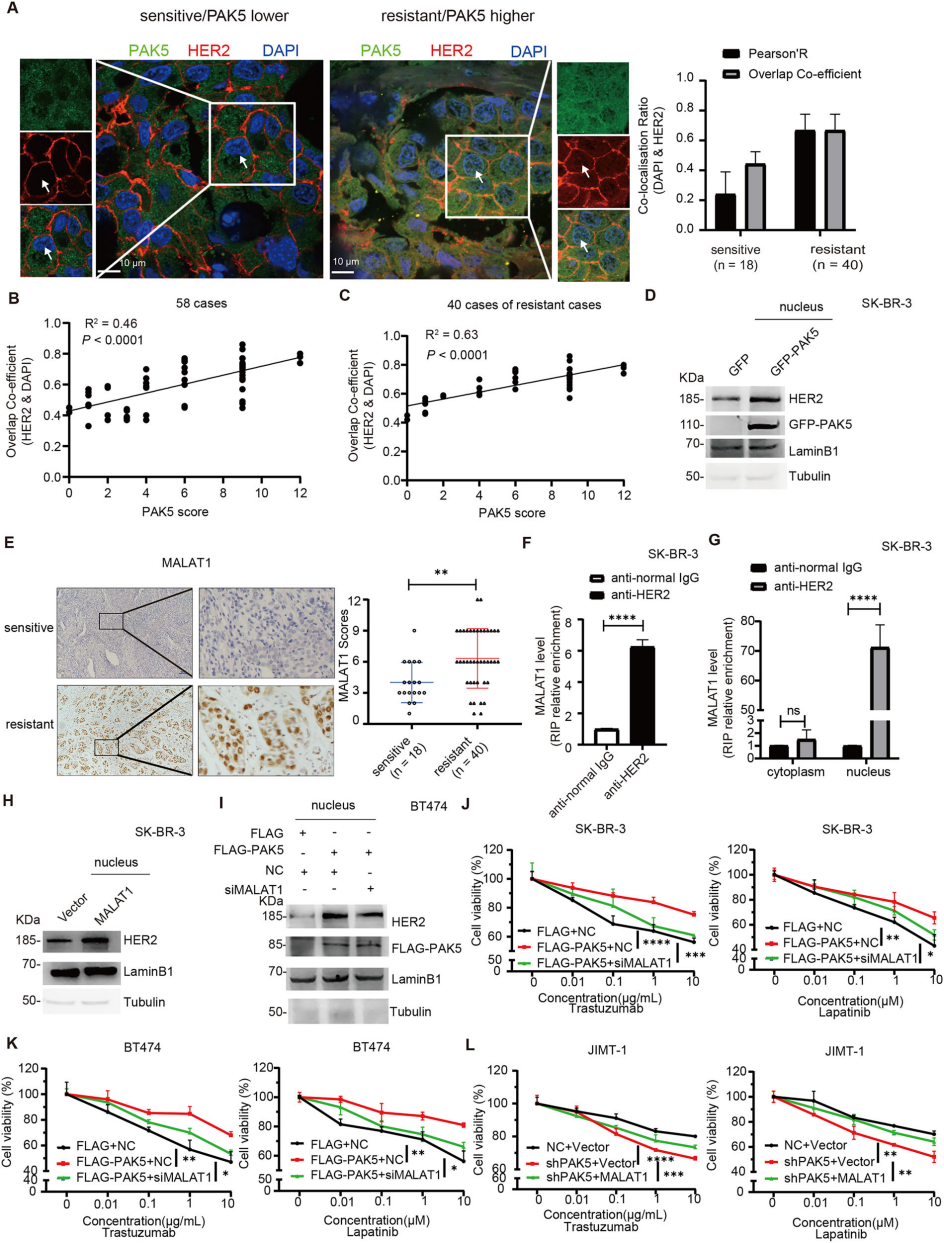
PAK5 increases the N-HER2 protein level via lncRNA MALAT1. **A** 18 cases of sensitive and 40 cases of resistant breast cancer tissues were collected for immunofluorescence analysis for HER2 (red). Nucleus was stained with DAPI. Original magnification was ×600. The Pearson’s correlation and Overlap Co-efficient were shown in bar graph format were analyzed (right). **B**, **C** Pearson’s correlation analysis of Overlap Co-efficient (HER2 & DAPI) and PAK5 scores. **B** all 58 cases, **C** 40 cases of resistant breast cancer tissues. **D** PAK5 increased the expression of N-HER2. SK-BR-3 cells were transfected with GFP vector or GFP-PAK5 and extracted nuclear protein. The relative level of nuclear protein was analyzed with LaminB1, and cytoplasmic protein with Tubulin (*n* = 3 biological replicates). **E** Representative images of MALAT1 staining by ISH analysis in breast cancer specimens treated with trastuzumab (left). The boxed areas in the left images were magnified in the right images. Original magnification, ×100. IHC staining scores for MALAT1 in HER2 positive breast cancer tissues from sensitive patients (*n* = 18) and resistant patients (*n* = 40) (right). **p < 0.01, *t*-test. **F** RIP analysis of the binding of HER2 and MALAT1. Enriching MALAT1 using anti-HER2 antibody and control IgG in SK-BR-3 cells (*n* = 3 biological replicates). ****p < 0.0001, *t*-test. **G** RIP analysis of the binding of HER2 and MALAT1 by cytoplasmic and nuclear protein extraction. Enriching MALAT1 using anti-HER2 antibody and control IgG in SK-BR-3 cells (*n* = 3 biological replicates). ns, *p* > 0.05, ****p < 0.0001, *t*-test. **H** MALAT1 increased the protein level of N-HER2. SK-BR-3 cells were transfected with vector or MALAT1 and extracted nuclear protein. The relative level of nuclear protein was analyzed with LaminB1, and cytoplasmic protein with Tubulin (*n* = 3 biological replicates). **I** PAK5 increased the protein level of N-HER2 though MALAT1. BT474 cells stably expressing FLAG vector or FLAG-PAK5 were transfected with MALAT1 siRNA or control vector, and extracted nuclear protein. The relative level of nuclear protein was analyzed with LaminB1, and cytoplasmic protein with Tubulin (*n* = 3 biological replicates). SK-BR-3 (**J**) and BT474 (**K**) cells stably expressing FLAG vector or FLAG-PAK5 were transfected with MALAT1 siRNA or control vector, were treated with trastuzumab or lapatinib at indicated concentrations for 72 h, cell viability was evaluated by CCK8 assay (*n* = 3 biological replicates). *p < 0.05, **p < 0.01, ***p < 0.001, ****p < 0.0001, one-way ANOVA. **L** JIMT-1 cells stably expressing PAK5 shRNA or control NC were transfected with vector or MALAT1, were treated with trastuzumab or lapatinib at indicated concentrations for 72 h, cell viability was evaluated by CCK8 assay (*n* = 3 biological replicates). **p < 0.01, ***p < 0.001, ****p < 0.0001, one-way ANOVA.

We further testified the relationship between the co-expression of HER2 and PAK5 and trastuzumab resistance in clinic. Excitingly, we found an increase in N-HER2 expression in patients with high PAK5 expression, who were resistant to trastuzumab treatment (Fig. 2A). Since N-HER2 is the major proliferation driver in trastuzumab-resistant breast cancer cells [8]. Prior to this, the clinical correlation between N-HER2 and trastuzumab resistance was still unclear. In addition, high expression of PAK5 was positively correlated with HER2 nuclear localization, especially with trastuzumab resistance (Fig. 2B, C). To determine the impact of PAK5 on HER2 nuclear expression, we overexpressed PAK5 and found that N-HER2 protein was increased significantly (Fig. 2D).

To delve into the mechanism of increased N-HER2 protein expression by PAK5, we utilized bioinformatics tools to predict proteins [29] (Table S2) and lncRNA [30] (Tables S3 and S4) that may interact with both PAK5 and HER2. Among them, a notably strong candidate with binding affinity to both was lncRNA MALAT1 which is reportedly related to HER2. We further verified the relationship between MALAT1 expression and trastuzumab resistance in clinic. ISH staining of MALAT1 in HER2 positive breast cancer patients (*n* = 58) treated with trastuzumab showed that patients with high expression of MALAT1 were resistant to trastuzumab treatment (Fig. 2E).

Since the current research showed that lncRNA MALAT1 was mainly located in the nucleus [31, 32], we wondered whether MALAT1 interacted with the N-HER2 protein. Firstly, we confirmed the interaction between MALAT1 and HER2 using RNA immunoprecipitation (RIP) and qRT-PCR assays (Fig. 2F) and this interaction between them occurred in the nucleus (Fig. 2G). Next, MALAT1 overexpression significantly decreased trastuzumab- and lapatinib-mediated growth inhibition in SK-BR-3 cells and BT474 cells (Fig. S2A, B), while MALAT1 silencing significantly increased the inhibition in JIMT-1 cells (Fig. S2C). We verified that overexpression of MALAT1 upregulated HER2 protein level, while silencing MALAT1 downregulated HER2 protein level (Fig. S2D, E). Lastly, we found that overexpression of MALAT1 really upregulated the level of N-HER2 protein (Figs. 2H and S2F). To further investigate whether PAK5 upregulates N-HER2 dependent on MALAT1, we confirmed that PAK5 upregulated N-HER2 protein level partly through MALAT1 (Figs. 2I and S2G). PAK5 significantly reduced the trastuzumab- and lapatinib-mediated growth inhibition via MALAT1 (Figs. 2J–L and S2G–I). Since HER2 overexpression activates Akt, leading to the downregulation of FOXO1, a negative regulator of MALAT1, by reducing its binding to the MALAT1 promoter. Consequently, HER2 upregulates MALAT1 expression [19]. We also confirmed that overexpression of HER2 upregulated the expression of PAK5, and MALAT1 (Fig. S2J,K). Importantly, PAK5 and HER2, especially N-HER2, were co-overexpressed in trastuzumab-resistant breast cancer tissues (Fig. 2A). Together with previous studies, there was a positive feedback loop between HER2 and PAK5. These data indicate that PAK5 increases the N-HER2 protein level via lncRNA MALAT1, which contributes to trastuzumab resistance in HER2-positive breast cancer.

### MALAT1 recruits deubiqutinase USP8 to inhibit N-HER2 ubiquitin proteasomal degradation and promotes N-HER2 accumulation

**Fig. 3:**
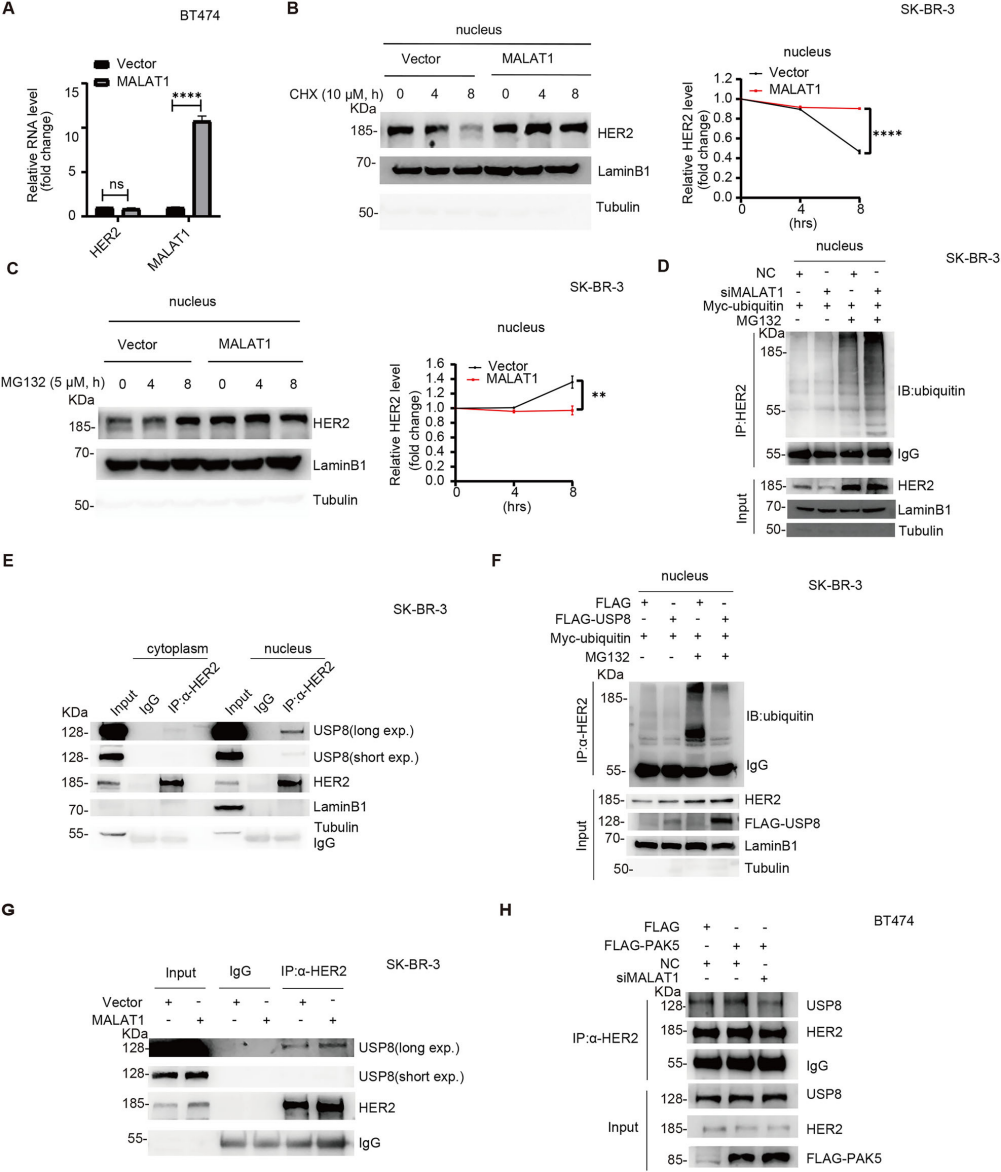
MALAT1 recruits deubiqutinase USP8 to inhibit N-HER2 ubiquitin proteasomal degradation and promotes N-HER2 accumulation. **A** qRT-PCR analysis of the mRNA level of HER2 in MALAT1 overexpressing cells (*n* = 3 biological replicates). ns, *p* > 0.05, ****p < 0.0001, *t*-test. IB analysis of the extracted nuclear proteins after CHX (**B**) or MG132 (**C**) treatment. Quantification of the band intensities in the left panel was shown as the means ± SEM. Band intensity was normalized to the LaminB1 intensity (*n* = 3 biological replicates). **p < 0.01, ****p < 0.0001, *t*-test. **D** IP and IB analysis of the ubiquitin for N-HER2 in SK-BR-3 cells transfected with MALAT1 siRNA or control vector in the presence of MG132 (*n* = 3 biological replicates). **E** IP and IB analysis of the interaction of HER2 and USP8 in SK-BR-3 cells by extracting cytoplasmic and nuclear protein. The relative level of nuclear protein was analyzed with LaminB1, and cytoplasmic protein with Tubulin (*n* = 3 biological replicates). **F** IP and IB analysis of the ubiquitin for N-HER2 in SK-BR-3 cells transfected with the indicated plasmids in the presence of MG132 (*n* = 3 biological replicates). **G** IP and IB analysis of the interaction of HER2 and USP8 in SK-BR-3 cells transfected with the indicated plasmids (*n* = 3 biological replicates). **H** BT474 cells expressing FLAG vector or FLAG-PAK5 were transfected with MALAT1 siRNA or control vector, IP and IB analysis of the interaction of HER2 and USP8 (*n* = 3 biological replicates).

To gain mechanistic insight into the function of the PAK5-MALAT1-nuclear HER2 in breast cancer, we wondered whether MALAT1 regulated HER2 mRNA. The result showed that overexpression of MALAT1 almost did not regulate the level of HER2 mRNA (Fig. 3A). In conjunction with the data that MALAT1 upregulated the level of HER2 protein (Fig. S2D, E), which suggested that MALAT1 might participate in the post-translational modification of HER2 protein. To test the effect of MALAT1 on HER2 stability, we first compared the half-life of HER2 by performing a time course assay following treatment with cycloheximide (CHX). HER2 was more abundant and more stable upon ectopic MALAT1 expression (Fig. S3A). We next exposed MALAT1-overexpressing cells to proteasome inhibitor MG132. The HER2 protein level was obviously accumulated upon ectopic vector expression but not ectopic MALAT1 expression (Fig. S3B). Overexpression of MALAT1 inhibited the proteasomal degradation of N-HER2 protein (Figs. 3B, C and S3C, D). Immunoprecipitation (IP) and immunoblot (IB) analysis of HER2 ubiquitination confirmed that MALAT1 inhibited the level of N-HER2 ubiquitination modification (Fig. 3D). These results indicate that MALAT1 upregulates the level of N-HER2 protein by inhibiting its ubiquitin proteasomal degradation.

Deubiquitinases are the reverse executors of ubiquitination [33, 34], which can dissociate ubiquitin from the targeted protein of ubiquitination to prevent protein degradation. Therefore, we predicted the potential deubiquitinases that HER2 might bind to through UbiBrowser 2.0 [35] (Fig. S3E), and combined with existing literature reports, MALAT1 might affect the process of substrate protein ubiquitination through the deubiquitinase USP8 [36]. Therefore, we selected USP8 for further experimental verification. The IP and IB analysis found that HER2 and USP8 bound each other, especially N-HER2 (Fig. 3E), and USP8 mainly inhibited N-HER2 ubiquitination (Fig. 3F). When MALAT1 was overexpressed, the interaction between HER2 and USP8 was increased (Fig. 3G). Further data confirmed that PAK5 enhanced the interaction between HER2 and USP8 via MALAT1 (Fig. 3H). These data suggest that MALAT1 recruits deubiqutinase USP8 to inhibit N-HER2 ubiquitin proteasomal degradation and promotes N-HER2 accumulation.

### PAK5 is the kinase for a novel substrate METTL14 mediating MALAT1 stability

**Fig. 4:**
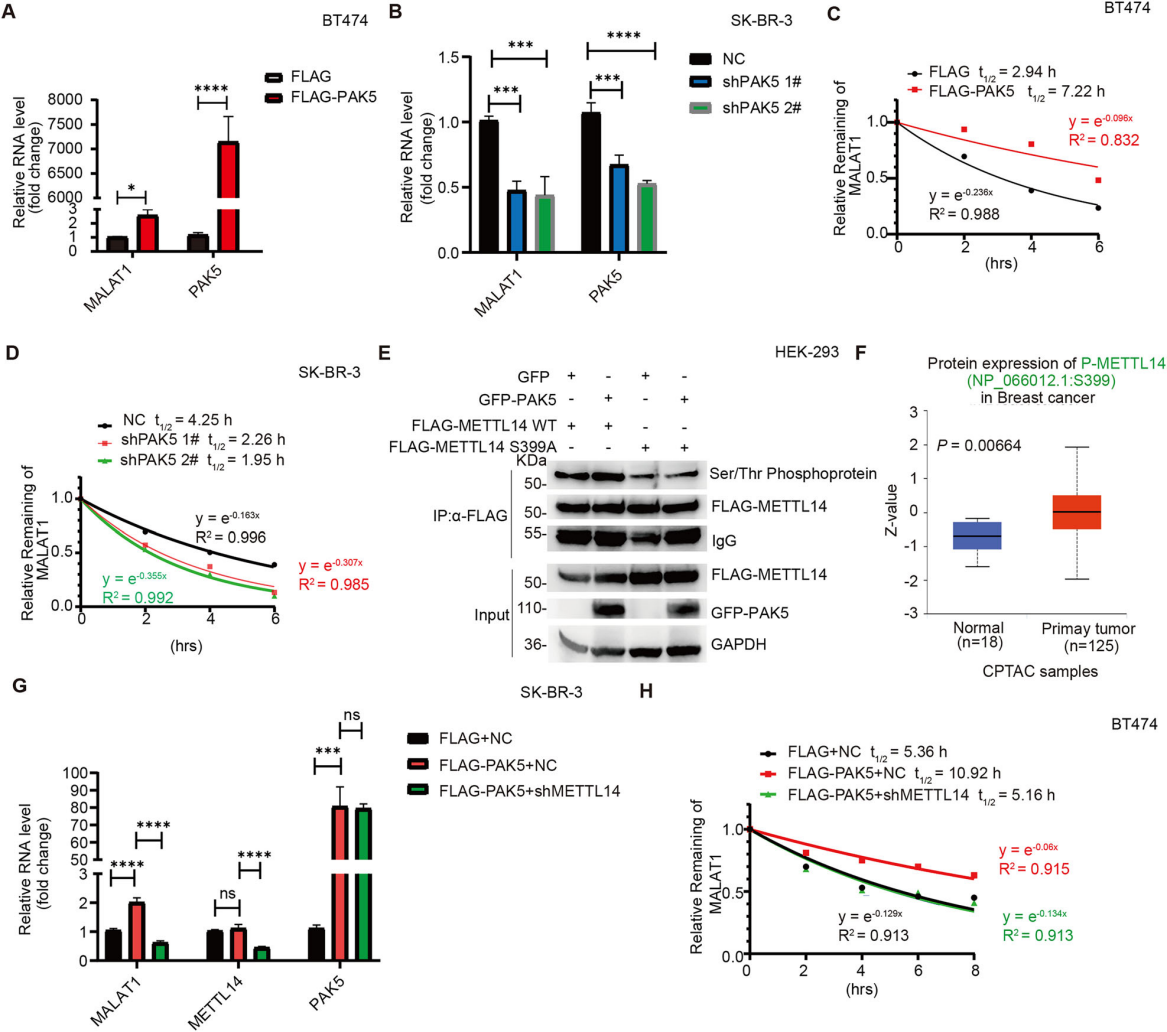
PAK5 is the kinase for a novel substrate METTL14 mediating MALAT1 stability. **A**, **B** qRT-PCR analysis of the RNA level of MALAT1 in PAK5 overexpressing cells (**A**, *t*-test) and PAK5-knowdown cells (**B**, one-way ANOVA) (*n* = 3 biological replicates). *p < 0.05, ***p < 0.001, ****p < 0.0001. **C**, **D** BT474 cells were transfected with FLAG or FLAG-PAK5, SK-BR-3 cells were transfected with NC or shPAK5 followed by treatment with actinomycin D (5 μg/mL) at the indicated time points, the RNA level of MALAT1 was detected by qRT-PCR assays (*n* = 3 biological replicates). **E** PAK5 phosphorylated the S399 site of METTL14. Lysates were immunoprecipitated with anti-FLAG antibody and immunoblotted with indicated antibodies (*n* = 3 biological replicates). **F** Analysis of METTL14 S399 phosphorylated protein expression in breast cancer and normal tissues with CPTAC database. **G** qRT-PCR analysis of the RNA level of MALAT1 in PAK5 overexpressing cells transfected with NC or shMETTL14 (*n* = 3 biological replicates). ns, *p* > 0.05, ***p < 0.001, ****p < 0.0001, one-way ANOVA. **H** BT474 cells were co-transfected with FLAG-PAK5 and shMETTL14 followed by treatment with actinomycin D (5 μg/mL) at the indicated time points, the RNA level of MALAT1 was detected by qRT-PCR assays (*n* = 3 biological replicates).

To explore how PAK5 regulate the MALAT1 expression, qRT-PCR assays revealed that overexpression of PAK5 upregulated the expression level of MALAT1 (Fig. 4A), while knocking down PAK5 also downregulated the expression level of MALAT1 (Fig. 4B). In addition, we treated BT474 and SK-BR-3 cells with actinomycin D, which restrained the transcription synthesis of RNA. The decay rate of lncRNA MALAT1 was decreased with PAK5 overexpression (Fig. 4C), and it was increased when PAK5 was silenced (Fig. 4D). These results indicated that PAK5 enhanced the stability of MALAT1 and upregulated MALAT1 expression.

N^6^-methyladenosine (m^6^A) modification [37, 38] plays a crucial role in the structural stability and expression level changes of lncRNA. PAK5 kinase acts on its targets mainly through phosphorylation and we overexpressed PAK5 in breast cancer cells and identified METTL14 with a phosphorylation site at serine 399 using phosphorylation mass spectrometry (Fig. S4A). Total Ser/Thr phosphorylated METTL14 protein from cell lysates was analyzed by immunoblotting. The results showed that phosphorylated wild-type METTL14 (METTL14 WT) but not METTL14 mutant (METTL14 S399A, site 399 serine was substituted with alanine, phosphorylation-disabled) was increased with overexpressing PAK5 (Fig. 4E), indicating that Ser399 on the METTL14 protein was phosphorylated by PAK5. We predicted the protein expression of METTL14 in breast cancer and adjacent cancer in the CPTAC database (https://ualcan.path.uab.edu/analysis-prot.html) and found that METTL14 was highly expressed in breast cancer (Fig. S4B) and phosphorylation of METTL14 S399 was also increased (Fig. 4F), suggesting that METTL14 phosphorylated at S399 may play an important role in the process of breast cancer.

METTL14, as a key component of the m^6^A methyltransferase complex [39, 40], catalyzes m^6^A modification on non-coding RNA to regulate its expression. We performed a coimmunoprecipitation (coIP) assay to validate the PAK5-METTL14 interaction. The interaction between GFP-tagged PAK5 (GFP-PAK5) and FLAG-tagged METTL14 (FLAG-METTL14) was observed in HEK-293 cells (Fig. S4C). In addition, the endogenous interaction of PAK5 with METTL14 was demonstrated in SK-BR-3 breast cancer cells (Fig. S4D). GST pulldown confirmed direct combination of PAK5 and METTL14 (Fig. S4E). Furthermore, PAK5 significantly colocalized with METTL14 in the nucleus (Fig. S4F). Next, METTL14 upregulated MALAT1 expression and enhanced MALAT1 RNA stability (Fig. S4G-S4I). We further verified whether PAK5 was involved in regulating the stability of MALAT1 mediated by METTL14. The results showed that PAK5 upregulated lncRNA MALAT1 expression and stability mediated by METTL14 (Fig. 4G, H). These data suggest that METTL14 is a novel phosphorylation substrate of PAK5 kinase, which mediates MALAT1 stability and expression.

### PAK5 promotes METTL14-mediated MALAT1 m^6^A modification

**Fig. 5:**
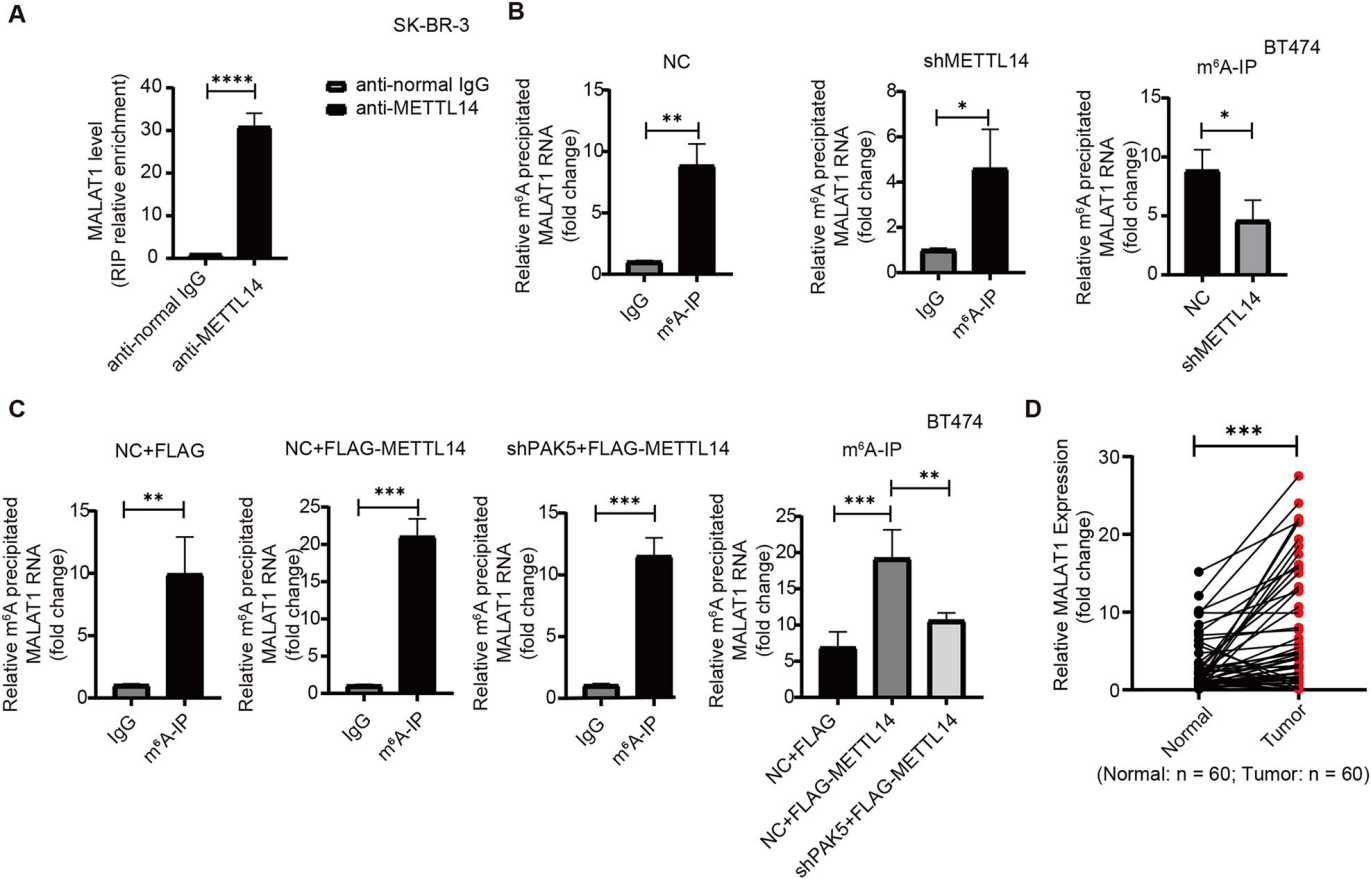
PAK5 promotes METTL14-mediated MALAT1 m^6^A modification. **A** RIP analysis of the binding of METTL14 and MALAT1. Enriching MALAT1 using anti-METTL14 antibody and control IgG in SK-BR-3 cells (*n* = 3 biological replicates). ****p < 0.0001, *t*-test. **B** BT474 cells were stably transfected with METTL14 shRNA or control shRNA, the m^6^A modification of MALAT1 were measured by MeRIP-PCR assays (*n* = 3 biological replicates). *p < 0.05, **p < 0.01, *t*-test. **C** BT474 cells were stably co-transfected with shPAK5 and METTL14, the m^6^A modification of MALAT1 was measured by MeRIP-PCR assays (*n* = 3 biological replicates). **p < 0.01, ***p < 0.001, one-way ANOVA. **D** qRT-PCR analysis of the expression level of MALAT1 in breast cancer (n = 3 biological replicates). ***p < 0.001, *t*-test.

To confirm that METTL14 was a methyltransferase of lncRNA MALAT1, we wondered whether there was an interaction between METTL14 and MALAT1. The RIP analysis showed the binding of METTL14 to MALAT1 in breast cancer cells (Fig. 5A) and the MeRIP-PCR assays indicated that METTL14 knockdown significantly reduced m^6^A modification of MALAT1 in BT474 cells (Figs. 5B and S5). We further verified whether PAK5 was involved in METTL14-mediated MALAT1 m^6^A modification. The MeRIP-PCR assays showed that overexpression of METTL14 promoted MALAT1 m^6^A modification, while silencing PAK5 attenuated METTL14-mediated MALAT1 m^6^A modification (Fig. 5C). Meanwhile, we collected 60 pairs of breast cancer and its paired adjacent fresh tissues. qRT-PCR assays confirmed that MALAT1 also showed higher expression in breast cancer tissues than adjacent tissues (Fig. 5D). These data suggests that METTL14 functions as a methyltransferase of MALAT1, leading to elevated MALAT1 expression dependent on PAK5 in breast cancer.

### PAK5 phosphorylates METTL14 to enhance N-HER2 accumulation mediated by lncRNA MALAT1

**Fig. 6:**
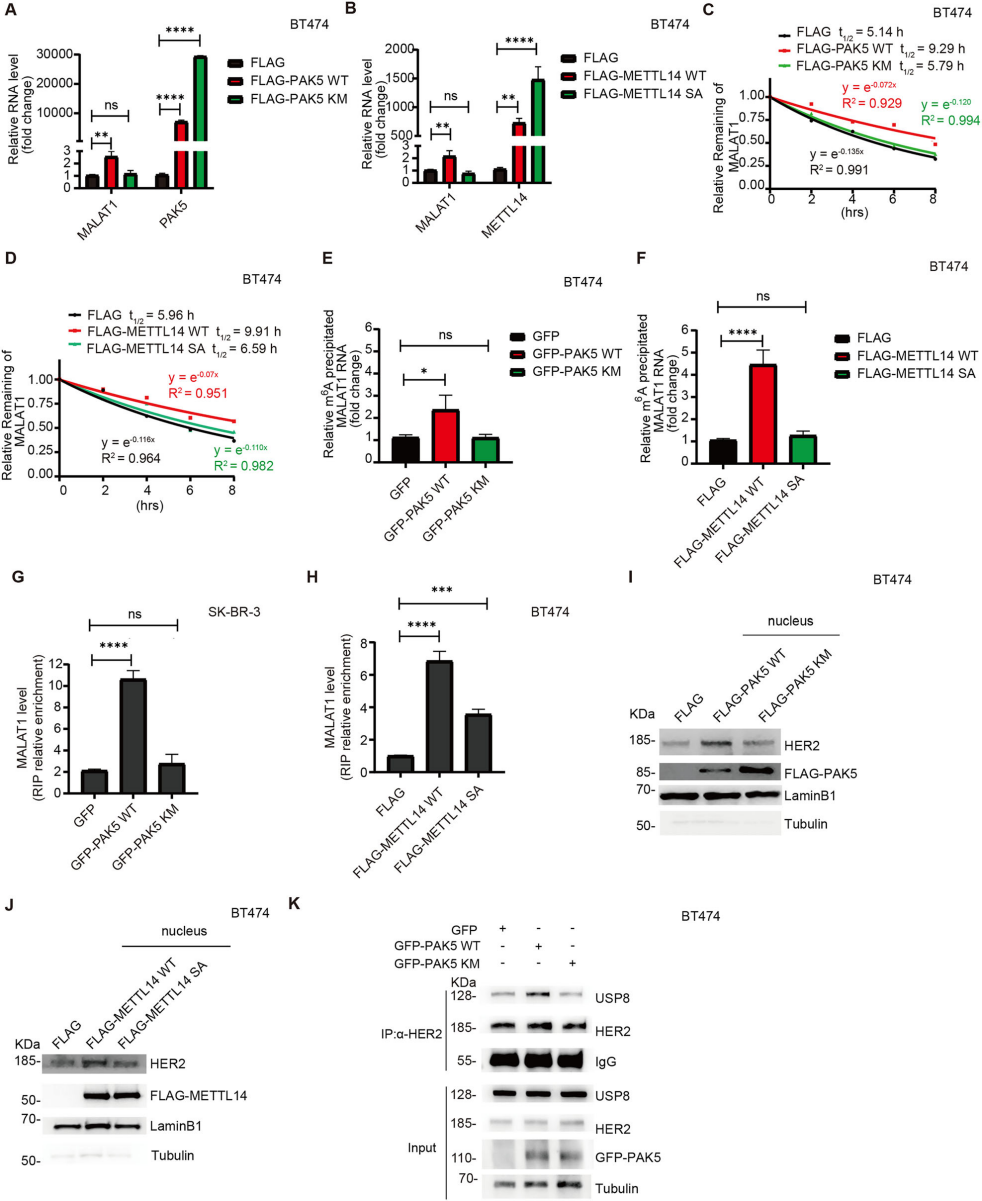
PAK5 phosphorylates METTL14 to enhance N-HER2 accumulation mediated by lncRNA MALAT1. qRT-PCR analysis of the expression level of MALAT1 in BT474 cells transfected with the PAK5 WT or PAK5 KM plasmid (**A**), METTL14 WT or METTL14 SA plasmid (**B**) (*n* = 3 biological replicates). ns, *p* > 0.05, **p < 0.01, ****p < 0.0001, one-way ANOVA. BT474 cells were transfected with the PAK5 WT or PAK5 KM plasmid (**C**), METTL14 WT or METTL14 SA plasmid (**D**) followed by treatment with actinomycin D (5 μg/mL) at the indicated time points, the RNA level of MALAT1 was detected by qRT-PCR assays (*n* = 3 biological replicates). BT474 cells were transfected with PAK5 WT or PAK5 KM plasmid (**E**), METTL14 WT or METTL14 SA plasmid (**F**), the m^6^A modification of MALAT1 was measured by MeRIP-PCR assays (*n* = 3 biological replicates). ns, *p* > 0.05, *p < 0.05, ****p < 0.0001, one-way ANOVA. **G** RIP analysis of the binding of METTL14 and MALAT1. SK-BR-3 cells were transfected with the PAK5 WT or PAK5 KM plasmid, followed by using anti-METTL14 antibody for IP and qRT-PCR (*n* = 3 biological replicates). ns, *p* > 0.05, ****p < 0.0001, one-way ANOVA. **H** RIP analysis of the binding of METTL14 and MALAT1. BT474 cells were transfected with the METTL14 WT or METTL14 SA plasmid, followed by using anti-FLAG antibody for IP and qRT-PCR (*n* = 3 biological replicates). ***p < 0.001, ****p < 0.0001, one-way ANOVA. BT474 cells were transfected with PAK5 WT or PAK5 KM plasmid (**I**), METTL14 WT or METTL14 SA plasmid (**J**), and extracted nuclear protein. The relative level of nuclear protein was analyzed with LaminB1, and cytoplasmic protein with Tubulin (*n* = 3 biological replicates). **K** IP and IB analysis of the interaction of HER2 and USP8 in BT474 cells transfected with the indicated plasmids (*n* = 3 biological replicates).

To further determine whether METTL14 regulation of lncRNA MALAT1 depended on the phosphorylation of METTL14 by PAK5. We transfected wild-type PAK5 (PAK5 WT) or kinase-dead PAK5 (PAK5 KM; PAK5 KM equate to PAK5 K478M) into breast cancer cells. MALAT1 was upregulated upon ectopic PAK5 WT expression but not ectopic PAK5 KM expression (Fig. 6A). Consistently, MALAT1 was upregulated upon ectopic wild-type METTL14 (METTL14 WT) expression but not ectopic nonphosphorylatable METTL14 (METTL14 S399A or METTL14 SA) expression (Fig. 6B).

Then, the breast cancer cells were treated with actinomycin D to observe the stability of lncRNA MALAT1. The results confirmed that both PAK5 activation and PAK5-mediated METTL14 phosphorylation enhanced the stability of MALAT1 (Figs. 6C, D and S6A, B). PAK5 WT, but not PAK5 KM, markedly increased m^6^A modification level of MALAT1 (Fig. 6E). METTL14 WT also promoted MALAT1 m^6^A modification, while METTL14 SA did not (Fig. 6F). Furthermore, the interaction between METTL14 and MALAT1 was strengthened by PAK5 WT, but not PAK5 KM (Fig. 6G). METTL14 WT also bound with more MALAT1 than METTL14 SA (Fig. 6H). In addition, we examined how PAK5 WT and PAK5 KM regulate the nuclear level of HER2 protein. The results showed that PAK5 WT upregulated N-HER2 protein expression, but PAK5 KM had almost no effect (Fig. 6I). Simultaneously, METTL14 WT upregulated N-HER2 protein expression, whereas METTL14 SA did not (Fig. 6J), indicating that N-HER2 expression depended on the kinase activity of PAK5. We also found that PAK5 WT but not PAK5 KM enhanced the interaction between deubiquitinase USP8 and N-HER2 (Fig. 6K). These results suggest that PAK5 phosphorylates METTL14 to enhance the stability of lncRNA MALAT1, thereby promoting the recruitment of deubiquitinase USP8 to facilitate N-HER2 accumulation.

### PAK5 promotes the trastuzumab resistance of breast cancer cells by increasing N-HER2 protein in mice

**Fig. 7:**
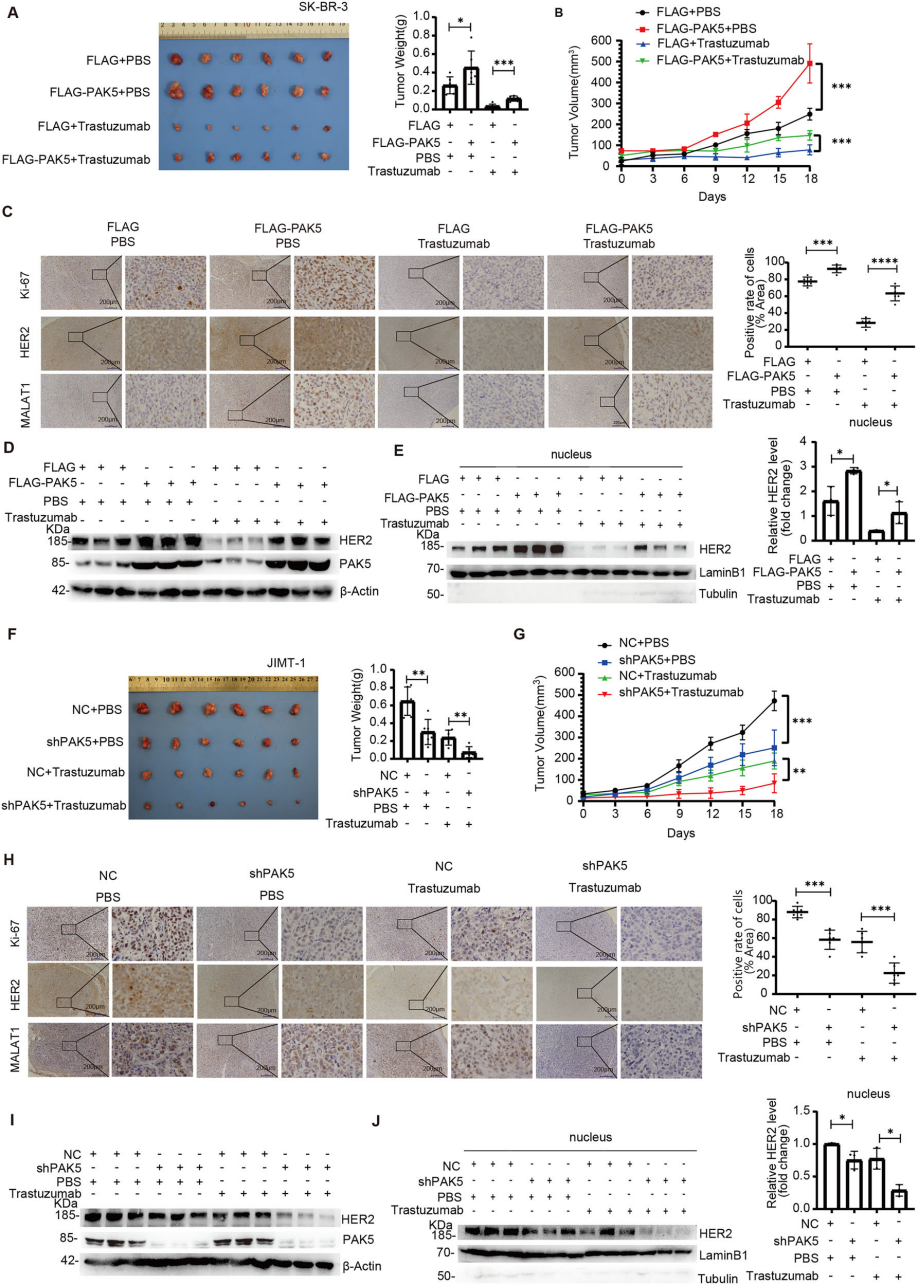
PAK5 promotes the trastuzumab resistance of breast cancer cells by increasing N-HER2 protein in mice. **A** At the end of treatment, tumors were excised and imaged. Quantitation of tumor weights was presented as a histogram (6 mice per group). *p < 0.05, ***p < 0.001, *t*-test. **B** The tumor size was measured at indicated time intervals and calculated, and growth curves were plotted using average tumor volume within each experimental group at the set time points (6 mice per group). ***p < 0.001, *t*-test. **C** Representative images of Ki-67, HER2 and MALAT1 IHC or ISH staining (left). Original magnification, ×100. All staining was quantified (6 mice per group) (right). ***p < 0.001, ****p < 0.0001, *t*-test. **D** Validation of HER2 and PAK5 protein levels in above tumors by IB (3 mice per group). **E** Validation of N-HER2 protein level in above tumors by IB (3 mice per group). *p < 0.05, *t*-test. **F** At the end of treatment, tumors were excised and imaged. Quantitation of tumor weights was presented as a histogram (6 mice per group). **p < 0.01, *t*-test. **G** The tumor size was measured at indicated time intervals and calculated, and growth curves were plotted using average tumor volume within each experimental group at the set time points (6 mice per group). **p < 0.01, ***p < 0.001, *t*-test. **H** Representative images of Ki-67, HER2 and MALAT1 IHC or ISH staining (left). Original magnification, ×100. All IHC staining was quantified (6 mice per group) (right). ***p < 0.001. **I** Validation of HER2 and PAK5 protein levels in above tumors by IB (3 mice per group). **J** Validation of N-HER2 protein level in above tumors by IB (3 mice per group). *p < 0.05, *t*-test.

To further explore the potential effects of PAK5 on the growth of trastuzumab-resistant breast cancer, female nude mice were injected with PAK5-overexpression SK-BR-3 or control SK-BR-3 cells into their mammary gland fat pads to induce xenograft tumors. Once the tumors reached a size of 100 mm^3^, the tumor-bearing mice were randomized and injected intraperitoneally with the PBS as the control or trastuzumab, then their tumor growth was monitored continuously. Consistent with our in vitro results, specific overexpression of PAK5 significantly alleviated the inhibitory effects of trastuzumab on SK-BR-3 tumor growth, evidenced by a significant increment of tumor size and weight as compared to the FLAG control (Fig. 7A, B). IHC and ISH exhibited that Ki-67, HER2 and MALAT1 expression levels were decreased with the treatment of trastuzumab (Fig. 7C). In addition, compared with FLAG control group treated with trastuzumab, Ki-67 protein expression was obviously increased in the PAK5-overexpression SK-BR-3 tumors after treatment of trastuzumab (Fig. 7C). Meanwhile, whether or not treated with trastuzumab, the relative levels of HER2 protein and MALAT1 RNA were elevated in SK-BR-3 tumors overexpressing PAK5 compared to the FLAG control group (Fig. 7C). Immunoblot revealed that compared with the SK-BR-3 control tumors, the relative levels of PAK5, total HER2 (Fig. 7D) and N-HER2 protein (Fig. 7E) were increased in the PAK5-overexpression SK-BR-3 tumors. These data further supported that PAK5 attenuated inhibitory role of trastuzumab treatment by stabilizing the N-HER2 protein in SK-BR-3 tumors. We further validated the drug treatment response in mice using trastuzumab-resistant JIMT-1 cells. Consistently, compared with the control group, treatment with trastuzumab significantly inhibited the growth of PAK5-silenced JIMT-1 tumors (Fig. 7F, G). IHC exhibited that Ki-67 expression was decreased with the trastuzumab treatment (Fig. 7H). Importantly, the expression levels of Ki-67, HER2 protein and MALAT1 RNA were obviously decreased in the PAK5-silenced JIMT-1 tumors after trastuzumab treatment as compared to NC control group with trastuzumab treatment (Fig. 7H). Finally, compared with the JIMT-1 control tumors, the relative levels of PAK5, total HER2 (Fig. 7I) and N-HER2 protein (Fig. 7J) were reduced in the PAK5-silenced JIMT-1 tumors after trastuzumab treatment. These data further support the notion that PAK5 promotes trastuzumab resistance in HER2 positive breast cancer by enhancing N-HER2 accumulation.

**Fig. 8:**
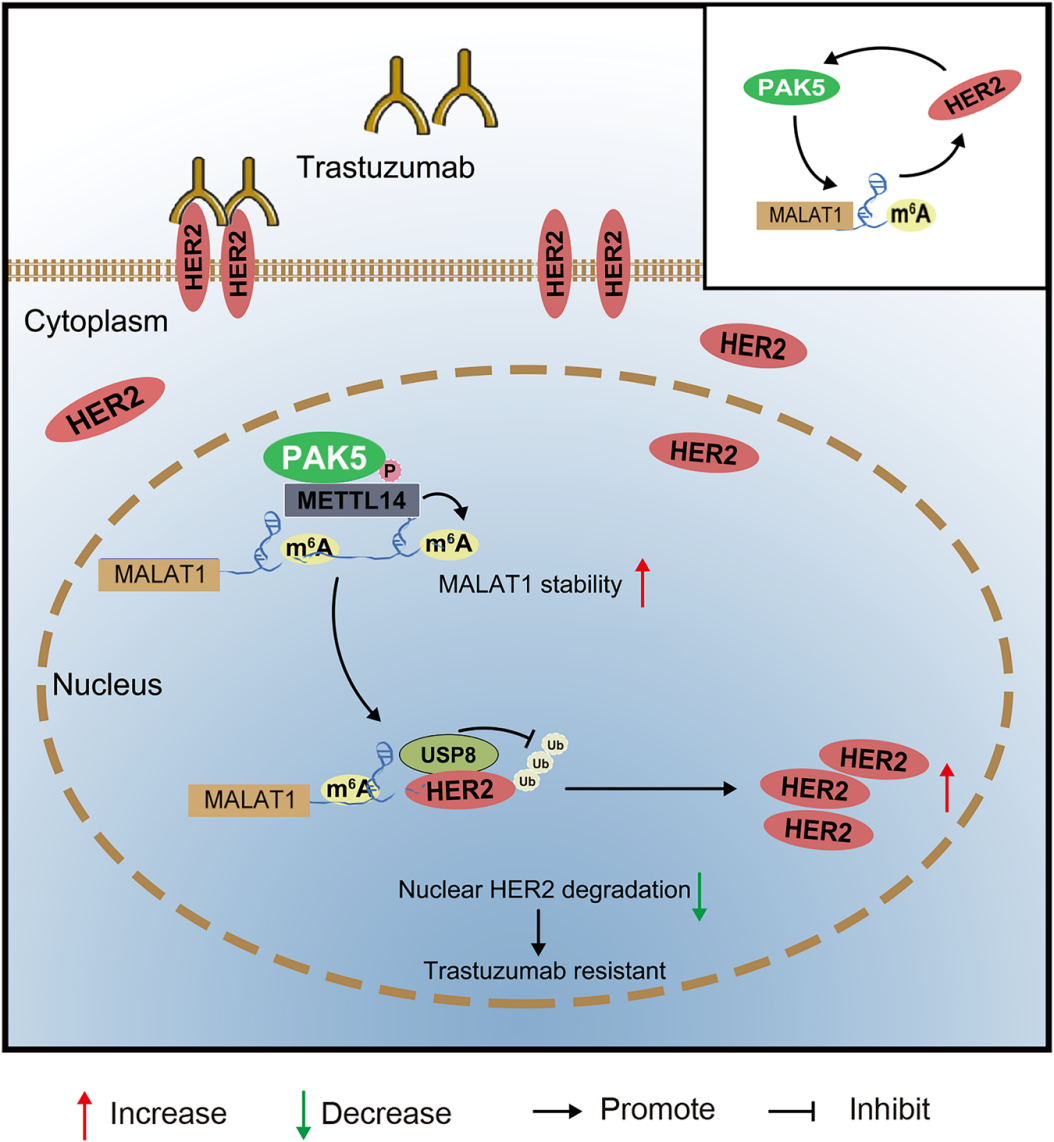
PAK5 promotes the trastuzumab resistance by increasing HER2 nuclear accumulation in HER2-positive breast cancer. PAK5 phosphorylates METTL14 to promote MALAT1 m^6^A modification and its stability; MALAT1 recruits USP8 to inhibit N-HER2 degradation and promote N-HER2 accumulation; PAK5 promotes the trastuzumab resistance of breast cancer cells by increasing N-HER2 protein in mice.

Based on the prior work and our own discoveries, we propose a hypothetical model. The high expression of PAK5 is associated with HER2-targeted therapy resistance and poor outcomes of breast cancer patients. In HER2-positive breast cancer, elevated expression of PAK5 promotes m^6^A modification of lncRNA MALAT1 through phosphorylation of the methyltransferase METTL14, thereby stabilizing MALAT1 RNA level. The increased expression of MALAT1 enhances the binding of deubiquitinase USP8 to N-HER2, which inhibits the N-HER2 ubiquitin proteasomal degradation, leading to the N-HER2 accumulation. This process may play a key role in resistance to trastuzumab therapies in HER2-positive breast cancer (Fig. 8).

## Discussion

Nowadays anti-HER2 monoclonal antibody such as trastuzumab greatly improves the survival of HER2-positive breast cancer patients [41]. However, 25-30% of HER2-positive breast cancer patients still suffer recurrence after standard treatment [42], and the mechanisms of trastuzumab resistance remain largely unclear. This study evaluated the role of PAK5 in trastuzumab resistance and explored the potential mechanisms underlying the action of PAK5 in trastuzumab resistance for HER2-positive breast cancer. Here, we have found that the expression of PAK5 was elevated in clinical HER2-positive breast cancer had poor response to treatment with trastuzumab, which was associated with poor prognosis in breast cancer patients. Additionally, PAK5 inhibits the therapeutic response of HER2-positive breast cancer cells to trastuzumab in vitro and in vivo. These results demonstrate that PAK5 may be involved in trastuzumab resistance of HER2-positive breast cancer.

Several mechanisms of resistance to trastuzumab have been uncovered in HER2-positive breast cancer, including the loss of trastuzumab binding site at the HER2 extracellular domain [43], PIK3CA mutation [44], PTEN loss [45], overexpression of RTKs [46], as well as the inhibitions of antibody-dependent cell-mediated cytotoxicity and antibody-dependent cellular phagocytosis [2]. Our results indicate that N-HER2 increases in clinical trastuzumab-resistant tissues. N-HER2 serves as a driving factor for resistance to monoclonal antibodies such as trastuzumab [8]. Nuclear translocation of HER2 can lead to a loss of drug target sites, resulting in ‘off-target’ effect, additionally, N-HER2 functions within the nucleus to promote cell proliferation and migration, thereby enhancing cellular trastuzumab resistance [47–49]. Furthermore, N-HER2 predominantly exists in tumor tissues and cells with higher malignancy levels [50]. Thus, N-HER2 is considered an independent prognostic factor for poor overall survival in HER2-positive breast cancer patients [6].

MALAT1 induces EMT and cell invasion and promotes HER2-positive cells to become resistant to trastuzumab treatment [20]. Here, we display that PAK5 promotes MALAT1-mediated N-HER2 accumulation. In HER2-overexpressing cells, HER2 dimerizes with its co-receptor HER3 which, in turn, directly couples to the p85 regulatory subunit of PI3K and activates the PI3K-AKT survival pathway [21]. Furthermore, activation of Akt by phosphorylation is associated with the upregulation of MALAT1. The transcription factor FOXO1 regulates the expression of MALAT1 via the PI3/Akt pathway. Consequently, HER2 upregulates MALAT1 expression. The results from our study also confirmed that overexpression of HER2 upregulated the expression of MALAT1. PAK5 promotes the recruitment of USP8 for N-HER2 to inhibit N-HER2 ubiquitination degradation through lncRNA MALAT1, leading to N-HER2 accumulation. Together with previous studies, there was a positive feedback loop between HER2 and PAK5.

The ubiquitin-proteasome pathway (UPP) is involved in the degradation of more than 80% of proteins in cells [51, 52]. Ubiquitination and deubiquitination have been observed to be dysregulated in various types of cancers [53, 54]. USP8 is recruited to the EGFR via multiple interactions including binding of the USP8 DUB domain to ubiquitinated EGFR and recruitment of USP8 to the EGFR on endosomal membranes via the USP8 N-terminal domain [27]. We delve into the mechanism and find that MALAT1 promotes the binding USP8 to N-HER2. PAK5 promotes the recruitment of USP8 for N-HER2 to inhibit N-HER2 ubiquitination degradation thought lncRNA MALAT1, leading to N-HER2 accumulation.

N^6^-methyladenosine (m^6^A) modifications, the most prevalent form of epigenetic modification in RNA, intricately linked to cell proliferation, metastasis, metabolism, and therapeutic resistance [22]. M^6^A modification affects most aspects of RNA regulation, such as alternative polyadenylation, splicing, nuclear exportation, stability and translation initiation [55]. As the key component of the m^6^A modification complex, METTL14 catalyzes m^6^A modification on mRNA or non-coding RNA to regulate its expression [25]. Our results indicate that PAK5 phosphorylates METTL14 at serine 399 and the phosphorylation of METTL14 S399 site in breast cancer tissues is more active than that in normal tissues. The phosphorylated METTL14 by PAK5 combines more MALAT1, leading to more stable expression of MALAT1. In HER2-positive breast cancer, elevated expression of PAK5 promotes m^6^A modification of lncRNA MALAT1 through phosphorylation of the methyltransferase METTL14 at serine 399, thereby stabilizing MALAT1 RNA level. The upregulated MALAT1 enhances the binding between USP8 and N-HER2, inhibiting the N-HER2 ubiquitin proteasomal degradation and promoting N-HER2 accumulation, PAK5 induces HER2 accumulation by inhibiting N-HER2 ubiquitination degradation via stabilization of MALAT1, which occurs through PAK5-mediated phosphorylation of METTL14, thereby playing a crucial role in breast cancer targeted trastuzumab resistance.

Although previous results suggest promoting function for PAK5 in breast cancer, definitive evidence related to drug resistance in breast cancer has been lacking until this study was performed. Our data establish that overexpressed PAK5 promotes the phosphorylation of the methyltransferase METTL14, facilitating m^6^A modification of lncRNA MALAT1 and stabilizing MALAT1 RNA level. The upregulated MALAT1, additionally, affects the binding between the deubiquitinase USP8 and N-HER2, inhibits the N-HER2 ubiquitin proteasomal degradation and promotes N-HER2 accumulation. This mechanism plays an important role in resistance to targeted therapy in HER2-positive breast cancer.

In conclusion, this study establishes, for the first time, the connection between PAK family members and lncRNA m^6^A modification. It systematically elucidates the mechanism of N-HER2 accumulation in HER2-positive breast cancer targeted therapy resistance. The study opens a potential therapeutic avenue for the treatment of HER2-positive breast cancer.

## Methods

### Cell lines and cell culture

Human breast cancer cell lines BT474 and SK-BR-3 were purchased from the Chinese Academy of Sciences Cell Bank (Shanghai). Human breast cancer cell line JIMT-1 was a gift from Dr.Liu Caigang (PMID: 36627608). Human embryonic kidney cell line HEK-293 was purchased from American Type Culture Collection (ATCC). The human breast cancer cell line BT474 was cultured in RPMI-1640 medium, HEK-293 in DMEM medium and SK-BR-3 in McCoy'5 A medium. Above cell culture media were supplemented with 10% FBS. JIMT-1 in DMEM medium was supplemented with 20% FBS. All cells were co-transfected with the constructed plasmids using Lipofectamine 3000 (Invitrogen) according to the manufacturer’s instructions.

### Animal model

Nude mice without thymus (approximately 4–5 weeks old) were purchased from SiPeiFu (SiPeiFu, Beijing, China). All experiments involving mice were carried out in accordance with the National Institutes of Health Guide for the Care and Use of Laboratory Animals (NIH Publication No.8023, revised 1978). All mice were maintained and bred under specific pathogen-free conditions at the Animal Center of China Medical University. Animal experiments were approved by the Animal Center of China Medical University and Committees of China Medical University. To evaluate the effect of PAK5 on the sensitivity of trastuzumab, 5 × 10^6^ SK-BR-3 FLAG and 5 × 10^6^ SK-BR-3 FLAG-PAK5 cells, 5 × 10^6^ JIMT-1 NC and 5 × 10^6^ JIMT-1 shPAK5 cells suspended in 100 μL PBS, with 50 μL Matrix gel (BD Biosciences) were mixed and injected into the fat pad of female thymus free mice to generate xenograft tumors. When the tumor size reaches ~100 mm^3^, mice were randomly divided and intraperitoneally (i.p.) injected with trastuzumab (10 mg/kg) or PBS as a control (*n* = 6) to evaluate the effect of PAK5 on HER2-targeted therapy resistance. The treatment was administered every other day, and the tumor volume calculation formula is: volume = π/6 × (length × width^2^), where length is the longest axis and width is a measurement value at right angles to length. At the end of treatment, the mice were euthanized and the tumors were collected for further analysis.

### Clinical samples

Our study included two independent experimental cohorts. The first cohort included 102 cases with fresh frozen tumors and adjacent tissues selected from September 2021 to September 2023 in Breast Surgery Department of the First Affiliated Hospital of China Medical University. Immunoblot was used to detect the expression of PAK5 protein of 102 pairs breast cancer tissues, and qRT-PCR assays was used to detect the expression of MALAT1 in 60 of 102 pairs breast cancer tissues.

The second group included 58 HER2 positive breast cancer patients from the Department of Medical Oncology of the First Affiliated Hospital of the China Medical University between 2009 and 2023. The molecular subtypes were determined by immunohistochemistry (IHC), and HER2 status was further confirmed by fluorescence in situ hybridization (FISH) if the IHC result was intermediate positive. All these patients received anti-HER2 treatment after surgery were divided into trastuzumab-resistant group and trastuzumab-sensitive group according to Guidelines for breast cancer diagnosis and treatment by China Anti-cancer Association (2024 edition) [56]. In addition, all patients experienced disease progression within 12 months of adjuvant trastuzumab treatment. Disease-free survival (DFS) was defined as the interval between the date of diagnosis and the first local or distant disease recurrence, or the last follow-up with relevant event. Overall survival (OS) was defined as the interval between the date of diagnosis and death caused by cancer.

The specimens were obtained with the written informed consent of all patients and were approved by the Ethics Committee of Affiliated Cancer Hospital & Institute of China Medical University. The study was conducted in accordance with the Declaration of Helsinki.

### Plasmid construction

The human PAK5 WT/K478M expression plasmids were gifts from Jonathan Chernoff (PMID: 16581795; PMID: 12897128). The human METTL14 expression plasmid was a gift from Dr.Yu Jianxiu (Shanghai Jiao Tong University, China (PMID: 29506078)). The human MALAT1 expression plasmid was purchased from Genechem (Shanghai, China). FLAG-METTL14 (WT, S399A mutation), FLAG-USP8, FLAG-HER2 and GST-METTL14 were amplified by PCR and constructed into pcDNA3.1 (invitrogen) and PGEX-4T-2 (GE Healthcare) vectors. The ubiquitin plasmid was previously generated in our laboratory.

### Lentiviral transfection

Human shPAK5 and shMETTL14 lentiviruses were purchased from Shanghai Genechem Co., Ltd. The human siMALAT1 were purchased from Sigma, and synthesized lentivirus by Shanghai Genechem Co., Ltd. According to the manufacturer’s instructions, cells were infected with lentivirus and infected cells were selected in puromycin (Sigma). The sequences were showed in Table S5.

### Immunoprecipitation, immunoblot and GST pulldown

For Immunoprecipitation (IP), cells were washed three times with an appropriate amount of pre-cooled PBS at 4 °C, and then lysed in IP lysis buffer containing proteasome inhibitors and protein phosphatase inhibitors. The Bradford method was used to measure the total protein in the whole cell lysate. Total protein lysate was immunoprecipitated with Protein A/G agarose beads (GE Healthcare Uppsala, Sweden) and specific antibodies, and incubated together overnight at 4 °C. The washed precipitated proteins were analyzed by IB. The IP, IB and GST pulldown used in this study had been described in detail previously [57].

### Cell immunofluorescence

Cells were fixed in 4% paraformaldehyde for 20 min at room temperature and then blocked with normal goat serum for 30 min at room temperature. Cells were washed three times in PBST (PBS containing 0.1% TritonX-100), incubated with PAK5 and METTL14 antibodies overnight at 4 °C, and subsequently secondary antibodies with red or green dye-labeled. The DNA was stained with the dye DAPI. Confocal scanning analysis was performed by using a Nikon laser confocal scanning microscope in accordance with established methods, utilizing sequential laser excitation to minimize the possibility of fluorescent emission bleed-through.

### Tissue Immunofluorescence

Tissues from 18 cases of sensitive and 40 cases of resistant breast cancer were blocked with goat serum, and incubated with the primary antibody HER2 (1:200; CST) overnight at 4 °C and subsequently incubated with secondary antibody conjugated with red dye for 40 minutes. Nucleus was stained using DAPI (Roche Applied Science) for 15 min. Overlap Co-efficient >0.5 is considered to enter the nucleus, and Overlap Co-efficient ≦0.5 is not entering the nucleus. Fluorescence microscope (Nikon, Tokyo, Japan) was used to acquire images.

### CCK8 assay

Cells (2 × 10^3^) were seeded in a 96-well plate, and CCK8 reagent was added at the indicated time points. After incubation at 37 °C for 2 h, the absorbance was detected at a wavelength of 450 nm. The cell proliferation ability was calculated according to the reagent instructions.

### RNA isolation and qRT-PCR

Total RNA from cells or tumors was extracted using the Trizol Reagent (Invitrogen, USA), and then was reverse transcribed using Prime Script™ RT Kit (Takara, China). The RNA expression level was detected with the SYBR premix ExTaqTM II kit (Takara, China) using the primers in Table S6. qRT-PCR assays was performed according to the previously used protocol [13] and the expression level of RNA relative to U6 was calculated by using Stratagene Mx 3000 P software (Agilent Technologies lnc., CA, USA).

### Immunohistochemical staining

Immunohistochemical staining was used to detect the expression of PAK5 (1:200; R&D) from 58 HER2-positive breast cancer patients who had received adjuvant trastuzumab treatment. The nucleus was stained by hematoxylin (Maixin Biotechnology Co. Ltd) as described previously [13]. After hydration and transparent, the sections were sealed with neutral resins and photographed. The staining intensity was graded as 0 (no color), 1 (light yellow), 2 (light brown), or 3 (brown), and the number of positive cells was graded as 0 (<5%), 1 (5–25%), 2 (25–50%), 3 (51–75%), or 4 (>75%). The two grades were multiplied. The score was 0-12. The H-score (histological score) was used to evaluate the staining results. We divided them into two groups: 0-4 was the low-expression group and 6-12 was the high-expression group.

### Cytoplasmic and nuclear protein extraction

Cytoplasmic and nuclear proteins were extracted using the Nucleus and Cytoplasmic Protein Extraction Kit (Beyotime, Shanghai, China). Briefly, the cells were collected after washing them with pre-cooled PBS, and added cytoplasmic separation reagent containing PMSF (proteasome inhibitor). After centrifugating, the supernatant was cytoplasmic protein, and the nuclei separation reagent was added into the pellet, and then complex was vortexed repeatedly for 10–15 times. After centrifugation again, the supernatant was nuclear protein. Finally, 30 μg of concentrated protein was used for immunoblot.

### RNA immunoprecipitation

RNA immunoprecipitation (RIP) was performed using the Magna RIP RBP immunoprecipitation kit (Millipore, 17-700) according to the manufacturer’s protocol. Briefly, breast cancer cells were washed 3 times with pre-cooled PBS, then lysed with RIP lysis buffer, and incubated with IgG or specific antibodies PAK5, GFP, FLAG, METTL14, HER2 and magnetic beads. For the magnetic bead-bound complexes, after unbound RNA was washed off, the precipitated RNA was purified, and using the standard qRT-PCR assays to detect RNA.

### RNA decay assay

Breast cancer cells were seeded in 6-well plates. After specific expression of different genes, actinomycin D (5 μg/mL) was used to treat cells at the indicated time points. Total RNA was isolated and qRT-PCR assays was performed to quantify the RNA with U6 as a reference.

### Methylated RNA immunoprecipitation (MeRIP)

Briefly, according to the manufacturer’s instructions, total RNA (300 μg) was extracted using Trizol and sheared into approximately 100 nt fragments by using the RNA fragmentation reagent in the kit (Millipore, 17-10499). After the anti-m^6^A antibody or normal IgG and the Magnetic beads were incubated at room temperature for 30 min, RNA fragmentation with the antibody-treated Magnetic beads was incubated overnight at 4 °C in immunoprecipitation buffer. After washing off the unbound RNA sufficiently, the precipitated RNA was extracted, purified and reverse transcribed into cDNA. The expression of MALAT1 was measured by qRT-PCR assays.

### Protein phosphorylation mass spectrometry

FLAG and FLAG-PAK5 were stably expressed in breast cancer cells. These cells were collected and digested used trypsin, then using a column containing affinity resin to enrich phosphorylated peptides, next a mass spectrometer to analyze phosphorylated peptides and determine phosphorylation sites (PTM BIO, Hangzhou, China).

### LncRNA ISH assay

RNA ISH assay was performed strictly following the kit instructions (Boster, Wuhan, China). Before prehybridized in prehybridization solution at 42 °C for 2 h, sections were deparaffinized and deproteinated, then incubated with the DIG-labeled probe solution (Dilute 4 times with 1×PBS) at 38 °C overnight. The specific sequences of probes for lncRNA MALAT1 were shown in Table S7. After stringent washing, the slides were exposed to a streptavidin-peroxidase reaction system and stained with DAB for 2 min. Then 0.1% Hematoxylin was used to counterstain the slides for 5 min. lncRNA MALAT1 expression levels were observed and counted under a microscope.

### Antibodies and reagents

Table S8 for details.

### Statistical analysis

The experiments were carried out at least 3 times, and the representative results were shown. All experimental results were expressed as mean ± SEM. For comparing the expression of MALAT1 or other targets between two groups, *p* values were calculated using two-tailed Student’s test. one-way ANOVA analysis was used to compare data between 3 groups. For the survival curves of patients between different groups, *p* values were calculated using log-rank testing. All statistical analyses used prism 8.0 (GraphPad Software Inc., La Jolla, CA). A value of *p* < 0.05 was considered statistically significant and *p* value < 0.05 was marked with (*), < 0.01 with (**), < 0.001 with (***) or < 0.0001 with (****).

## Supplementary information

Supplementary

Original western blots

## Data Availability

All data generated or analyzed during this study are included in this article (and its supplementary information files).
