# Supplementary material for: PAK5 promotes the trastuzumab resistance by increasing HER2 nuclear accumulation in HER2-positive breast cancer
Source: Cell Death Dis. 2025 Apr 21;16(1):323. doi: 10.1038/s41419-025-07657-2 (PMC12012021; doi:10.1038/s41419-025-07657-2)
Supplement: Supplementary file 1 — Supplementary [file 41419_2025_7657_MOESM1_ESM.docx]

**Supplementary Figure 1**


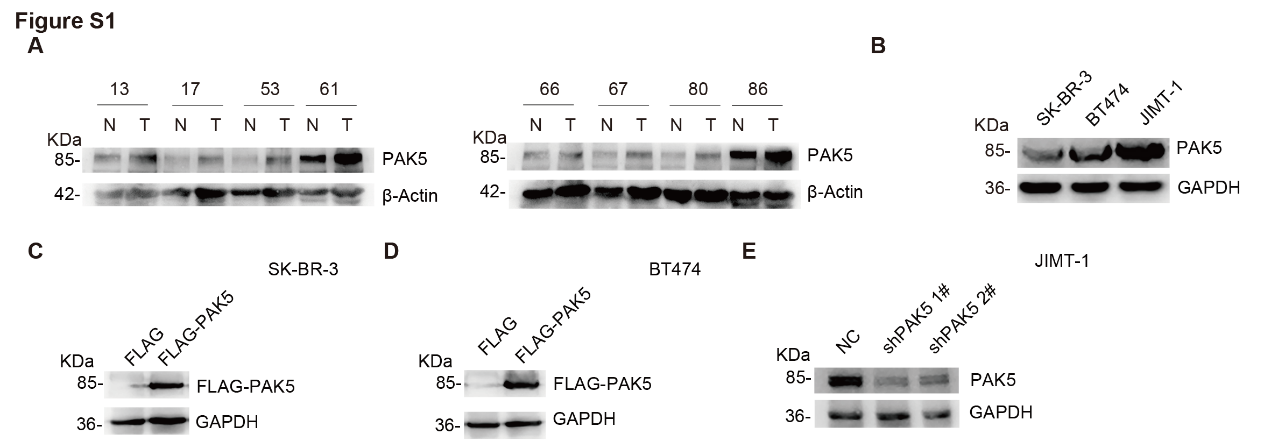


**Figure S1 A high level of PAK5 protein is associated with drug resistance in HER2 positive breast cancer patients receiving trastuzumab treatment. Related to Figure 1.** (A) The IB data were further analyzed to confirm the expression of PAK5 in breast cancer tissues. The representative eight pairs were shown. The relative protein level of PAK5 was analyzed with β-Actin. The lower/higher protein level in tumor tissues (T), compared with matched adjacent noncancerous tissues (N), is categorized as lower/higher protein level. Related to Table S1. (B) Validation of PAK5 expression in both HER2-targeted therapy sensitive cells (SK-BR-3 and BT474) and resistant cells (JIMT-1) by IB (n = 3 biological replicates). Related to Figure 1. (C-D) Construction of stable overexpression cell lines. FLAG vector or FLAG-PAK5 was stably expressed in SK-BR-3 and BT474 cells through lentivirus. Cell lysates from these cells were used for immunoblot using anti-FLAG and GAPDH antibodies. Related to Figure 1D-1E. (E) Construction of stable silent cell lines. NC and shPAK5 was stably expressed in JIMT-1 cells through lentivirus. Cell lysates from these cells were used for immunoblot using anti-PAK5 and GAPDH antibodies. Related to Figure 1F.

**Supplementary Figure 2**


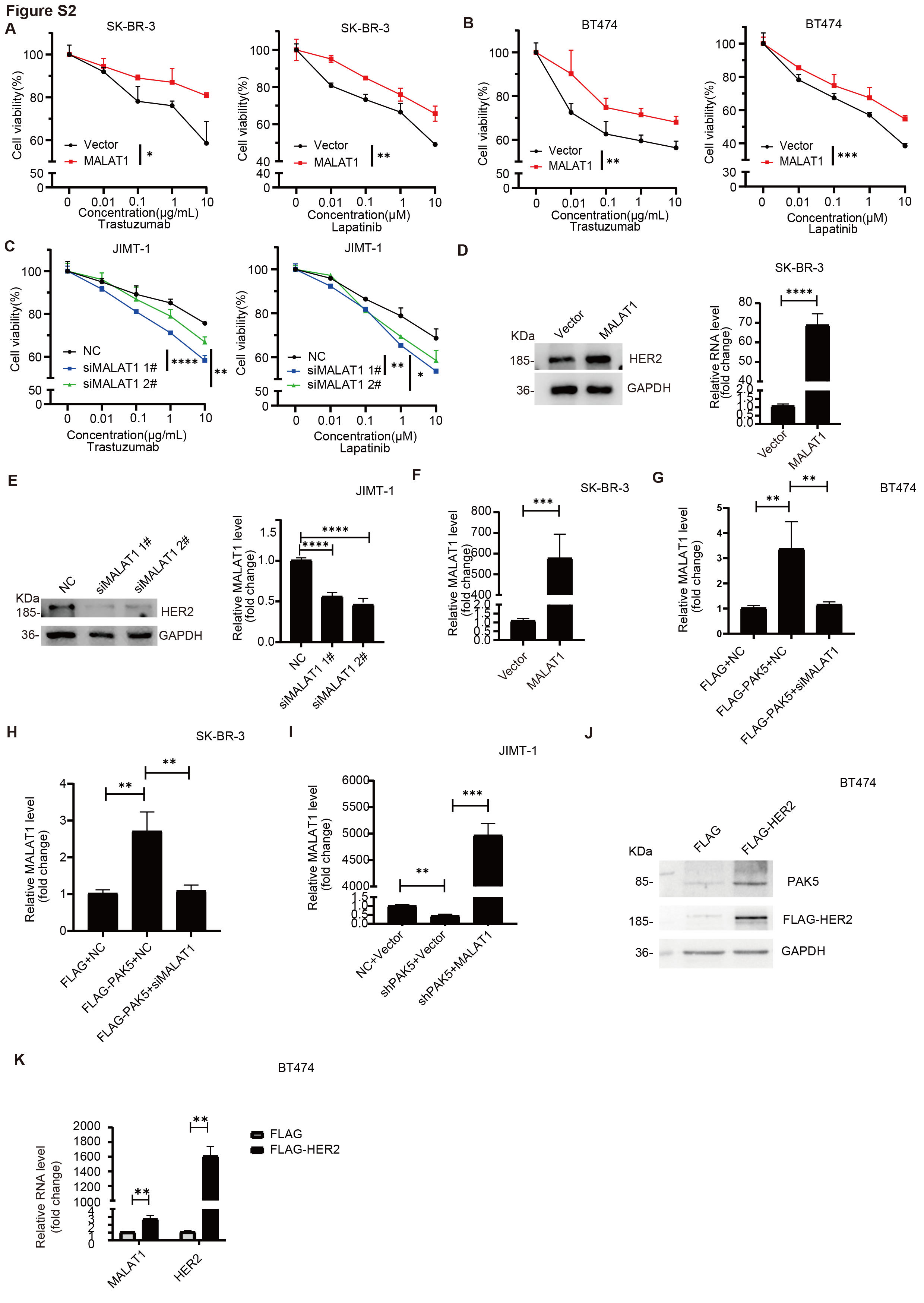


**Figure S2 PAK5 increases the N-HER2 protein level via lncRNA MALAT1. Related to Figure 2.** (A-B) SK-BR-3 (A) and BT474 (B) cells were transfected with MALAT1 plasmid, followed treating with trastuzumab or lapatinib at indicated concentrations for 72 h, cell viability was evaluated by CCK8 assay (n = 3 biological replicates). **p* < 0.05, ***p* < 0.01, ****p* < 0.001, t test. (C) JIMT-1 cells stably expressing MALAT1 siRNA or control NC were treated with trastuzumab or lapatinib at indicated concentrations for 72 h, cell viability was evaluated by CCK8 assay (n = 3 biological replicates). **p* < 0.05, ***p* < 0.01, *****p* < 0.0001, 1-way ANOVA. (D-E) IB analysis of the expression level of HER2 in MALAT1 overexpressing cells and MALAT1-knockdown cells (n = 3 biological replicates). *****p* < 0.0001. (F) SK-BR-3 cells were transfected with MALAT1 plasmid. ****p* < 0.001, t test. Related to Figure 2G. (G-H) BT474 (G) and SK-BR-3 (H) cells stably expressing FLAG vector or FLAG-PAK5 were transfected with MALAT1 siRNA or control vector. ***p* < 0.01, 1-way ANOVA. Related to Figure 2J-2K. (I) JIMT-1 cells stably expressing PAK5 shRNA or control NC were transfected with vector or MALAT1. ***p* < 0.01, ****p* < 0.001. Related to Figure 2L. (J) BT474 cells were transfected with the HER2. Cell lysate from these cells was used for immunoblot using anti-PAK5, FLAG and GAPDH antibodies. (K) qRT-PCR analysis of the RNA level of HER2 and MALAT1 in HER2 overexpressing cells (n = 3 biological replicates). ***p* < 0.01.

**Supplementary Figure 3**


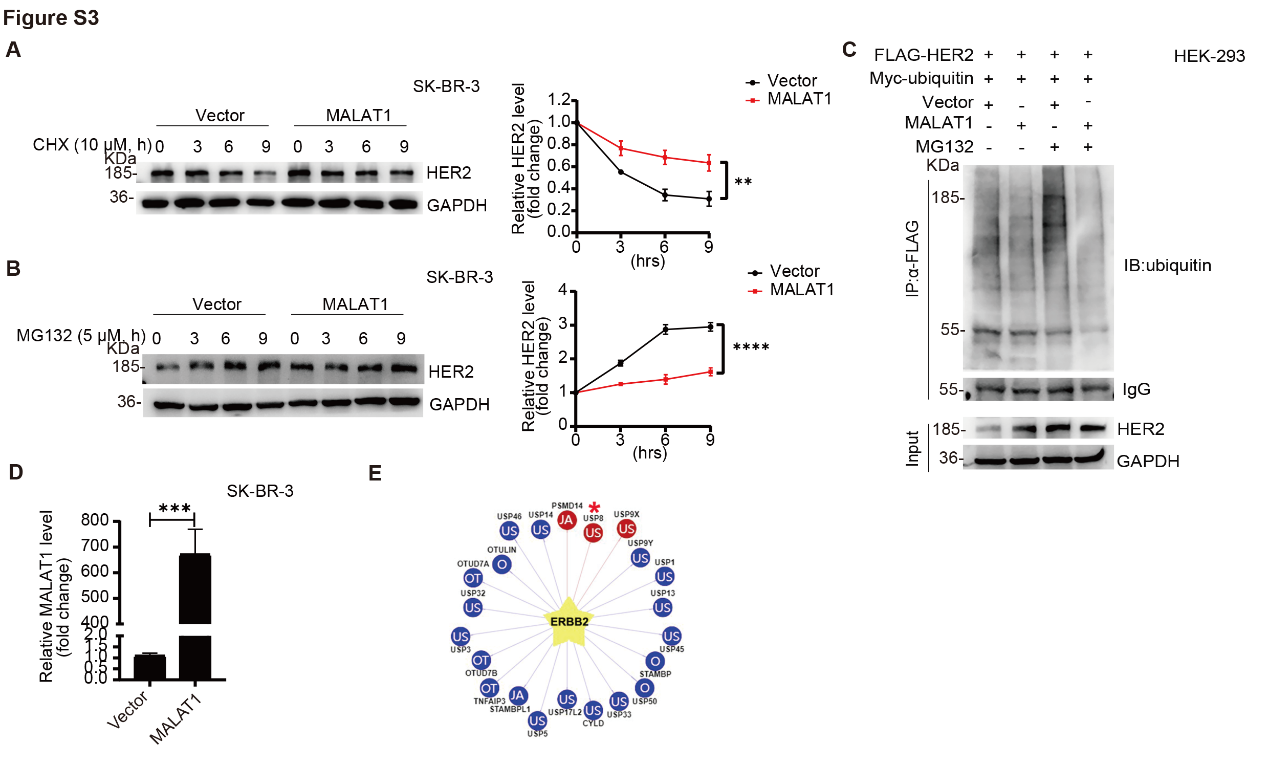


**Figure S3 MALAT1 recruits deubiqutinase USP8 to inhibit N-HER2 ubiquitin proteasomal degradation and promotes N-HER2 accumulation.** **Related to Figure 3.** (A-B) IB analysis of the extracted proteins after CHX or MG132 treatment. Quantification of the band intensities in the left panel was shown as the means ± SEM. Band intensity was normalized to GAPDH intensity (n = 3 biological replicates). ***p* < 0.01, *****p* < 0.0001, t test. (C) IP and IB analysis of the ubiquitin for HER2 in HEK-293 cells transfected with MALAT1 and FLAG-HER2 in the presence of MG132 (n = 3 biological replicates). (D) SK-BR-3 cells were transfected with MALAT1 plasmid. ****p* < 0.001, t test. Related to Figure 3B-3C. (E) Potential deubiquitinaes that HER2 may bind to through bioinformatics.

**Supplementary Figure 4**


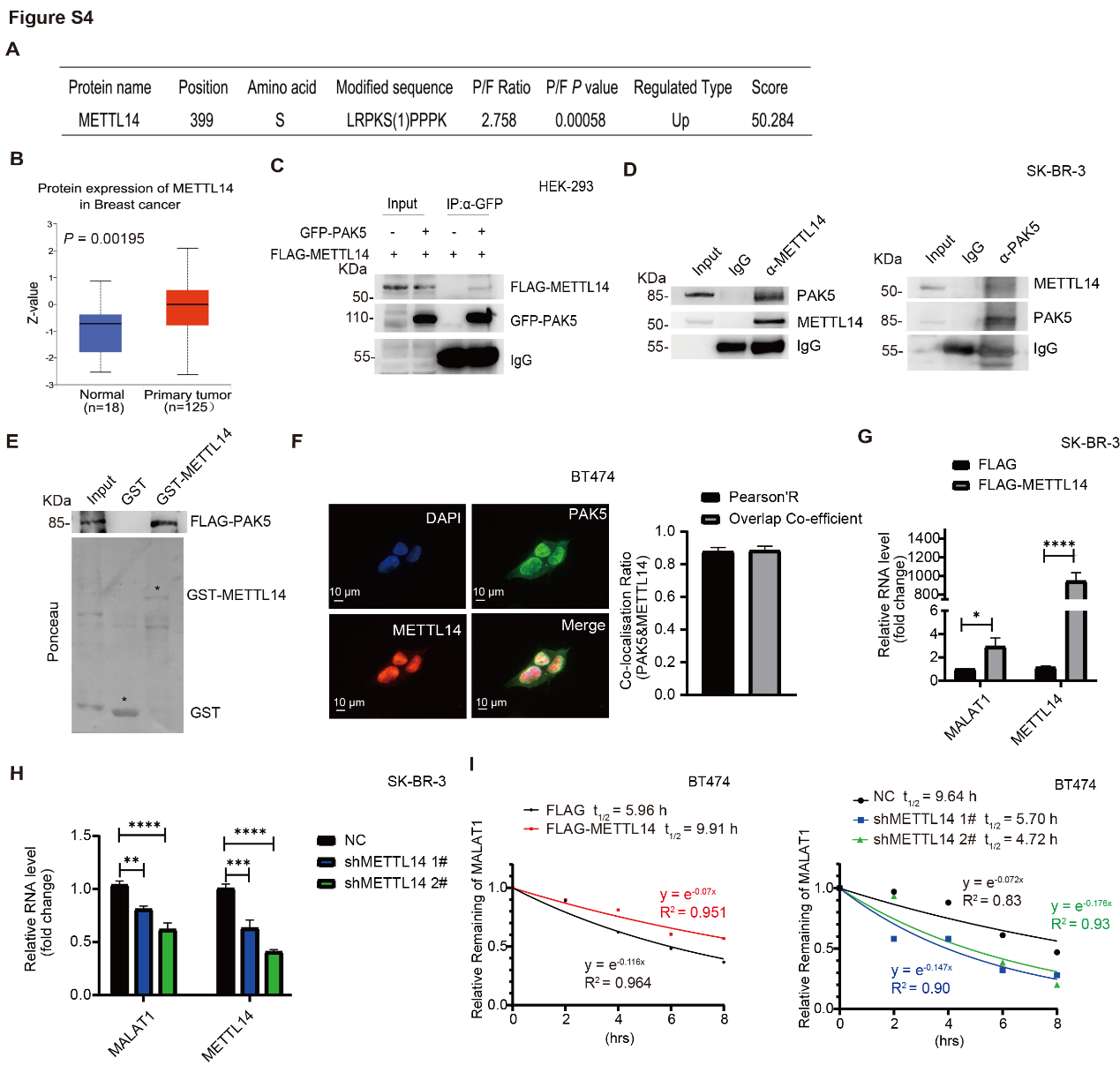


**Figure S4 PAK5 is the kinase for a novel substrate METTL14 mediating MALAT1 stability. Related to Figure 4.** (A) Phosphorylation quantitative mass spectrometry analysis in breast cancer cell lysate stably overexpressing FLAG and FLAG-PAK5. Related to Figure 4E. (B) Analysis of total METTL14 expression in breast cancer and normal tissues with CPTAC database. Related to Figure 4F. (C-D) IP and IB analysis of the interaction of exogenous GFP-PAK5 and FLAG-METTL14 in HEK-293 cells (C) and endogenous PAK5 and METTL14 in SK-BR-3 cells (D) (n = 3 biological replicates). (E) GST pulldown analysis of the direct binding of PAK5 and METTL14. Black stars indicate GST and GST-fusion proteins (n = 3 biological replicates). (F) Immunofluorescence staining of BT474 cells for PAK5 and METTL14 (left). Nucleus was stained with DAPI. Original magnification was 600 ×. The Pearson’s correlation and Overlap co-efficient (right) were shown in bar graph format from three independent experiments were analyzed (n = 3 biological replicates). (G-H) qRT-PCR analysis of the expression level of MALAT1 in METTL14 overexpressing cells (G) and METTL14-knowdown cells (H) (n = 3 biological replicates). **p* < 0.05, ***p* < 0.01, ****p* < 0.001, *****p* < 0.0001. (I) BT474 cells were transfected with FLAG or FLAG-METTL14 (left), and BT474 cells stably expressing METTL14 shRNA or control NC (right) followed by treatment with actinomycin D (5 μg/mL) at the indicated time points, the RNA level of MALAT1 was detected by qRT-PCR assays (n = 3 biological replicates).

**Supplementary Figure 5**


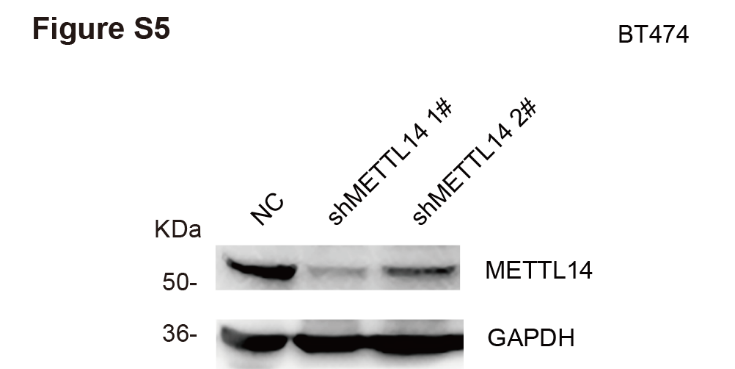


**Figure S5 PAK5 promotes METTL14-mediated MALAT1 m^6^A modification. Related to Figure 5.** Construction of stable silent cell lines. NC and shMETTL14 was stably expressed in BT474 cells through lentivirus. Cell lysate from these cells was used for immunoblot using anti-METTL14 and GAPDH antibodies (n = 3 biological replicates). Related to Figure 5B.

**Supplementary Figure 6**


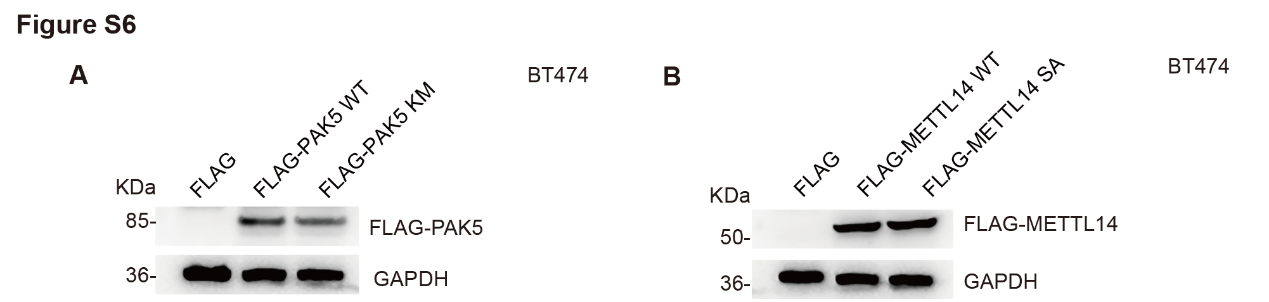


**Figure S6 PAK5 phosphorylates METTL14 to enhance N-HER2 accumulation mediated by lncRNA MALAT1. Related to Figure 6.** (A) BT474 cells were transfected with the PAK5 WT or PAK5 KM plasmid. Cell lysate from these cells was used for immunoblot using anti-FLAG and GAPDH antibodies. Related to Figure 6C. (B) BT474 cells were transfected with the METTL14 WT or METTL14 SA plasmid. Cell lysate from these cells was used for immunoblot using anti-FLAG and GAPDH antibodies. Related to Figure 6D.

**Supplementary Table 1**

**Table S1. PAK5 expression during breast cancer progression. Related to Figure 1**

| Factor | PAK5 expression | | *p* value |
| --- | --- | --- | --- |
|  | Low (n = 30) | High (n = 72) |  |
| Age |  |  | 0.6580 |
| <50 | 10 | 28 |  |
| ≥50 | 20 | 44 |  |
| Pathological stage (pStage) |  |  | 0.6607 |
| Stage Ⅰ-Ⅱ | 14 | 29 |  |
| Stage Ⅲ-Ⅳ | 16 | 43 |  |
| Lymph node metastasis(pN) |  |  | **0.0177*** |
| Yes | 2 | 21 |  |
| No | 28 | 51 |  |
| Distant metastasis(pM) |  |  |  |
| Yes | 3 | 23 | **0.0245*** |
| No | 27 | 49 |  |
| ER |  |  |  |
| Positive | 23 | 48 | 0.3550 |
| Negative | 7 | 24 |  |
| PR |  |  |  |
| Positive | 18 | 50 | 0.3668 |
| Negative | 12 | 22 |  |
| HER2 |  |  |  |
| Positive | 26 | 47 | **0.0321*** |
| Negative | 4 | 25 |  |

*Indicated statistical significance (*p* < 0.05)

**Supplementary Table 2**

**Table S2. Proteins interacting with PAK5 & HER2**

| Proteins interacting with PAK5 | Proteins interacting with HER2 | |
| --- | --- | --- |
| RAC1 | EGF | ERBB4 |
| ARHGEF6 | KRAS | GRB2 |
| PAK3 | PIK3CA | GRB7 |
| PAK1 | ERBB3 | HRAS |
| RAC3 | GAB1 | HSP90AA1 |
| PAK2 | CDC37 | HSP90AB1 |
| SRC | CBL | HSPA4 |
| PXN | PXN | HSPA8 |
| ARHGEF7 | MAPK3 | IGF1 |
| GIT2 | RAF1 | IRS1 |
| CTTN | AKT1 | ITGB3 |
| PAK4 | BTC | JAK2 |
| NCK1 | CD247 | MAP2K1 |
| CDC42 | EGFR | NF1 |
|  | RASA1 | NRAS |
|  | SHC1 | NRG1 |
|  | SHC3 | PIK3CG |
|  | SOS1 | PIK3R1 |
|  | VEGFA | PTPN11 |

**Supplementary Table 3**

**Table S3. LncRNA interacting with PAK5**

| RNA_ID | Top_RNA_fragment | | Interaction_Propensity | Ranking |
| --- | --- | --- | --- | --- |
| ENST00000501122_NEAT1 | 455-1364 | 317.05 | | 0.423833 |
| ENST00000429829_XIST | 1965-2734 | 160.31 | | 0.336542 |
| ENST00000563328_AC004943.2 | 1770-1963 | 137.29 | | 0.479125 |
| ENST00000623420_Z95114.4 | 454-597 | 136.71 | | 0.503333 |
| ENST00000534336_MALAT1 | 349-698 | 128.53 | | 0.404125 |
| ENST00000564460_AL008727.1 | 2190-2379 | 125.93 | | 0.460792 |
| ENST00000643862_AL121612.2 | 911-1094 | 124.65 | | 0.468833 |
| ENST00000444431_AL136982.1 | 1921-2104 | 122.29 | | 0.464833 |
| ENST00000565493_NORAD | 325-542 | 120.32 | | 0.452208 |
| ENST00000568885_AC027279.1 | 1701-1872 | 119.89 | | 0.477458 |
| ENST00000623569_UCKL1-AS1 | 3314-3459 | 118.31 | | 0.503333 |
| ENST00000623663_AL357673.2 | 1387-1586 | 117.64 | | 0.442333 |
| ENST00000630728_TARID | 777-972 | 117.5 | | 0.442125 |
| ENST00000568496_AC090826.1 | 3574-3771 | 115.24 | | 0.438625 |
| ENST00000620778_AC090340.1 | 1684-1883 | 114.74 | | 0.437833 |
| ENST00000659232_AL590822.3 | 161-322 | 114.66 | | 0.478167 |
| ENST00000451424_PINK1-AS | 353-530 | 113.86 | | 0.454875 |
| ENST00000611744_AL139385.1 | 2427-2558 | 113.51 | | 0.503333 |
| ENST00000445317_PRDM16-DT | 1009-1178 | 113.4 | | 0.47 |
| ENST00000488000_AC091230.1 | 3004-3187 | 111.86 | | 0.44725 |

**Supplementary Table 4**

**Table S4. LncRNA interacting with HER2**

| RNA_ID | Top_RNA_fragment | Interaction_Propensity | Ranking |
| --- | --- | --- | --- |
| ENST00000501122_NEAT1 | 455-1364 | 226.82 | 0.286 |
| ENST00000534336_MALAT1 | 349-698 | 125.7 | 0.323792 |
| ENST00000429829_XIST | 2689-3458 | 114.55 | 0.224708 |
| ENST00000564460_AL008727.1 | 2190-2379 | 113.17 | 0.363583 |
| ENST00000444431_AL136982.1 | 1921-2104 | 110.93 | 0.369042 |
| ENST00000451424_PINK1-AS | 353-530 | 108.1 | 0.3685 |
| ENST00000623420_Z95114.4 | 454-597 | 105.44 | 0.403 |
| ENST00000568885_AC027279.1 | 1701-1872 | 103.98 | 0.372417 |
| ENST00000609564_OGFRP1 | 2689-2882 | 100.27 | 0.342792 |
| ENST00000630728_TARID | 777-972 | 97.54 | 0.334458 |
| ENST00000427229_AL358394.2 | 901-1046 | 96.78 | 0.385875 |
| ENST00000563328_AC004943.2 | 1770-1963 | 95.98 | 0.335875 |
| ENST00000623663_AL357673.2 | 1387-1586 | 95.78 | 0.33175 |
| ENST00000568496_AC090826.1 | 1863-2060 | 95.72 | 0.331667 |
| ENST00000611744_AL139385.1 | 2427-2558 | 94.67 | 0.395042 |
| ENST00000669902_AC090994.1 | 1629-1718 | 94.57 | 0.426208 |
| ENST00000553181_ODC1-DT | 2521-2690 | 93.31 | 0.357042 |
| ENST00000607781_RN7SL832P | 71-142 | 92.6 | 0.426667 |
| ENST00000595909_AC008764.6 | 1559-1642 | 92.05 | 0.425958 |
| ENST00000560068_AC015712.2 | 1101-1230 | 91.65 | 0.388625 |

**Supplementary Table 5**

**Table S5. Sequence of shRNAs used in this study**

| METTL14 | shRNA 1# | **5’-**GCTGGACTTGGGATGATATTA-**3’** |
| --- | --- | --- |
|  | shRNA 2# | **5’-**GGAACAACTCAAGTAGCTTCA-**3’** |
| PAK5 | shRNA 1# | **5’-**CCGGATAAAGTTGTCTGATTT-**3’** |
|  | shRNA 2# | **5’-**CGGGATTACCACCATGACAAT-**3’** |
| MALAT1 | siRNA 1# | **5’-**GUGUUUAUGACCCGUUUADTDT  UAAACGGGUCAUCAAACACDTDT-**3’** |
|  | siRNA 2# | **5’-**CUUUCCACACGCUAGUAAUDTDT  AUUACUAGCGUGUGGAAAGDTDT-**3’** |

**Supplementary Table 6**

**Table S6. A list of primer sequence**

| **Primer name** | **Primer sequence (5’ to 3’)** |
| --- | --- |
| MALAT1 forward | GACGGAGGTTGAGATGAAGC |
| MALAT1 reverse | ATTCGGGGCTCTGTAGTCCT |
| PAK5 forward | AGAAGGTGGTGCCTTGACAGAC |
| PAK5 reverse | GTCCCTGTGAATCACTCCTTGG |
| METTL14 forward | TTTCTCTGGTGTGGTTCTGG |
| METTL14 reverse | AAGTCTTAGTCTTCCCAGGATTG |
| HER2 forward | TGCTGGACATTGACGAGACAGAGT |
| HER2 reverse | AGCTCCCACACAGTCACACCATAA |
| U6 forward | CTCGCTTCGGCAGCACA |
| U6 reverse | AACGCTTCACGAATTTGCGT |

**Supplementary Table 7**

**Table S7. Probes used in situ hybridization (ISH) assay**

| **Digoxin-labeled probe** | **sequence (5’ to 3’)** |
| --- | --- |
| MALAT1-(1) | AAAGTAAAGCCCTGAACTATCACACTTTAATCTTCCTTCA |
| MALAT1-(2) | AGAATTGCGTCATTTAAAGCCTAGTTAACGCATTTACTAA |
| MALAT1-(3) | GATTTATATGGGGACGTAGGCCGATTTCCGGGTGTTGTAG |

**Supplementary Table 8**

**Table S8. Antibodies and Reagents**

| **REAGENT or RESOURCE** | **SOURCE** | **IDENTIFIER** |
| --- | --- | --- |
| **Antibodies** |  |  |
| PAK5 | R&D | Cat# MAB4696; RRID: AB_2158766 |
| METTL14 | Proteintech | Cat# 26158-1-AP; RRID: AB_2800447 |
| HER2 | Cell Signaling Technology | Cat# 2165; RRID: AB_10692490 |
| FLAG-tag | GenScript | Cat# A00187; RRID: AB_1720813 |
| GFP-tag | GenScript | Cat# A00185; RRID: AB_914676 |
| Myc-tag | GenScript | Cat# A00704; RRID: AB_914461 |
| GAPDH | Kangchen Biotech | Cat# KC-5G4; RRID: AB_2493106 |
| β-Actin | Cell Signaling Technology | Cat# 3700; RRID: AB 2242334 |
| LaminB1 | Abcam | Cat# ab16048; RRID: AB_443298 |
| Tubulin | Abcam | Cat# ab7291; RRID: AB_2241126 |
| ubiquitin | Abcam | Cat# ab134953; RRID: AB_2801561 |
| Ki-67 | Abcam | Cat# ab16667; RRID: AB_302459 |
| Donkey anti-Mouse IgG (H+L) Highly Cross-Adsorbed Secondary Antibody, Alexa Fluor 488 | Thermo Fisher Scientific | Cat# A-21202; RRID: AB_141607 |
| Donkey anti-Rabbit IgG (H+L) Highly Cross-Adsorbed Secondary Antibody, Alexa Fluor 594 | Thermo Fisher Scientific | Cat# A-21207; RRID: AB_141637 |
| **Bacterial and virus strains** |  |  |
| DH5α Competent Cells | TaKaRa | 9057 |
| BL21 Competent Cells | TaKaRa | 9126 |
| Lentivirus | Genechem | N/A |
| **Chemicals** |  |  |
| CHX | MedChemExpress | HY-12320 |
| MG132 | MedChemExpress | HY-13259 |
| Actinomycin D | MedchemExprss | HY-17559 |
| Trastuzumab (anti-HER2) | Selleck | A2007 |
| Lapatinib | Selleck | S2111 |
| **Biological samples** |  |  |
| Human breast cancer tissues | The First Affiliated Hospital  of China Medical University | N/A |
| **Critical commercial assays** |  |  |
| Magna RIP RBP immunoprecipitation Kit | Millipore | 17-700 |
| the Pierce™ Magnetic RNA-Protein Pull-Down Kit | Thermo | 20164 |
| the Transcript Aid T7 High Yield RNA Synthesis Kit | Thermo | K0441 |
| Magna MeRIP™ m^6^A Kit- Transcriptome-wide Profiling of N^6^-Methyladenosine | Millipore | 17-10499 |
| TNT Quick Coupled Transcription/Translation System | Promega | L1170 |
| PrimeScript RT Reagent Kit | TaKaRa | RR037A |
| SYBRPremix Ex TaqII | TaKaRa | RR820A |
| **Experimental models: Cell lines** |  |  |
| BT474 | Shanghai cell bank of CAS-Chinese Academy of Sciences | TCHu143 |
| SK-BR-3 | Shanghai cell bank of CAS-Chinese Academy of Sciences | TCHu225 |
| JIMT-1 | donated | N/A |
| HEK-293 | ATCC | Cat# CRL-1573; RRID: CVCL_0045 |
| **Experimental models: Organisms/strains** | | |
| Mouse: BALB/c nude | Charles River | Strain Code: 401 |
| **Recombinant DNA** |  |  |
| FLAG-PAK5 WT | This paper | N/A |
| FLAG-PAK5 K478M | This paper | N/A |
| GFP-PAK5 WT | This paper | N/A |
| GFP-PAK5 K478M | This paper | N/A |
| FLAG-METTL14 WT | This paper | N/A |
| FLAG-METTL14 S399A | This paper | N/A |
| GST-METTL14 | This paper | N/A |
| FLAG-USP8 | This paper | N/A |
| Myc-ubiquitin | This paper | N/A |
| FLAG-HER2 | This paper | N/A |
| **Software and algorithms** |  |  |
| Prism | GraphPad | RRID:SCR_002798;  https://www.graphpad.com/ |
|  |  |  |
