# Supplementary figures and images for: PAK5 promotes the trastuzumab resistance by increasing HER2 nuclear accumulation in HER2-positive breast cancer
Source: Cell Death Dis. 2025 Apr 21;16(1):323. doi: 10.1038/s41419-025-07657-2 (PMC12012021; doi:10.1038/s41419-025-07657-2)

**2D**


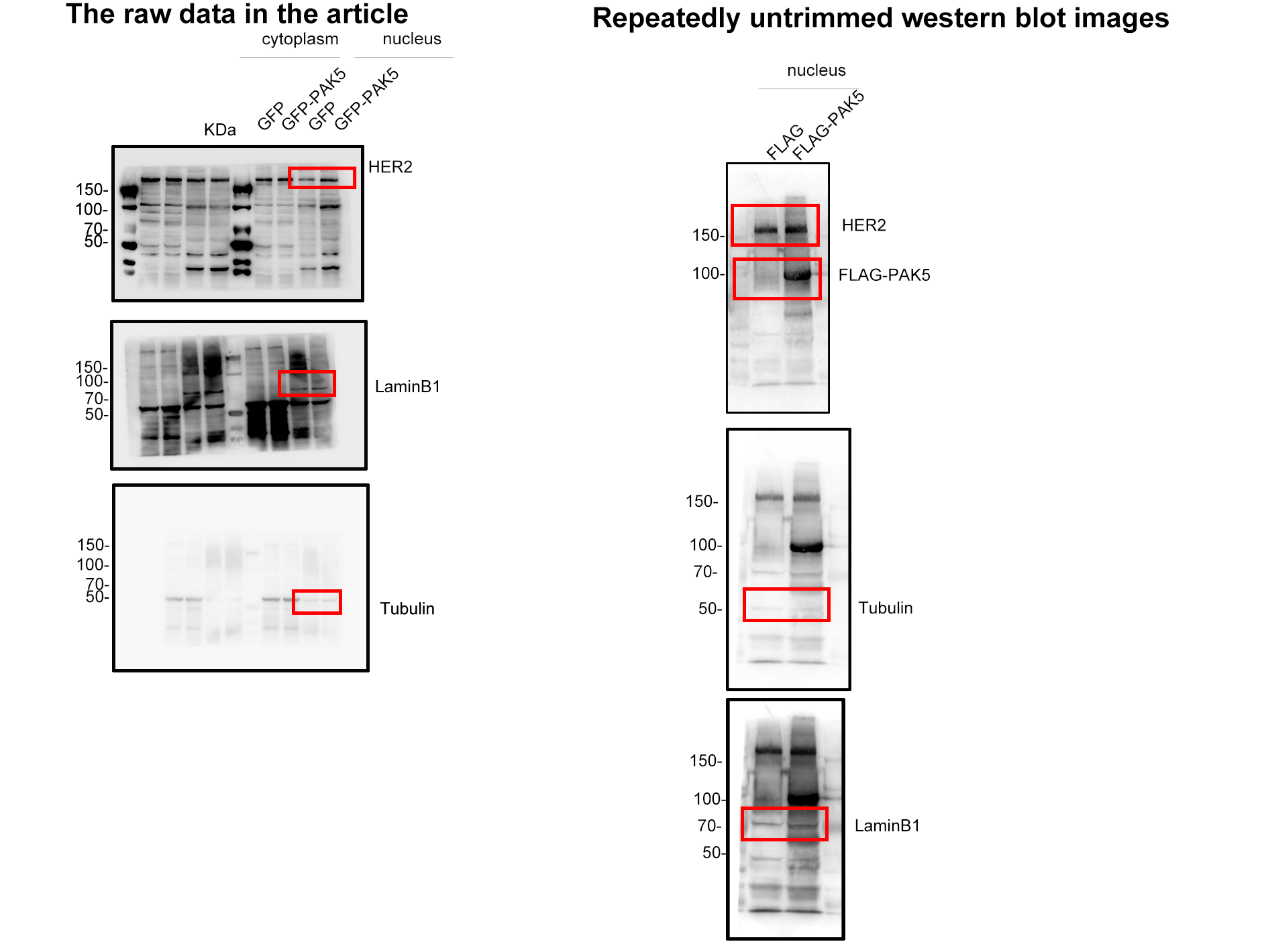


**2H**

**
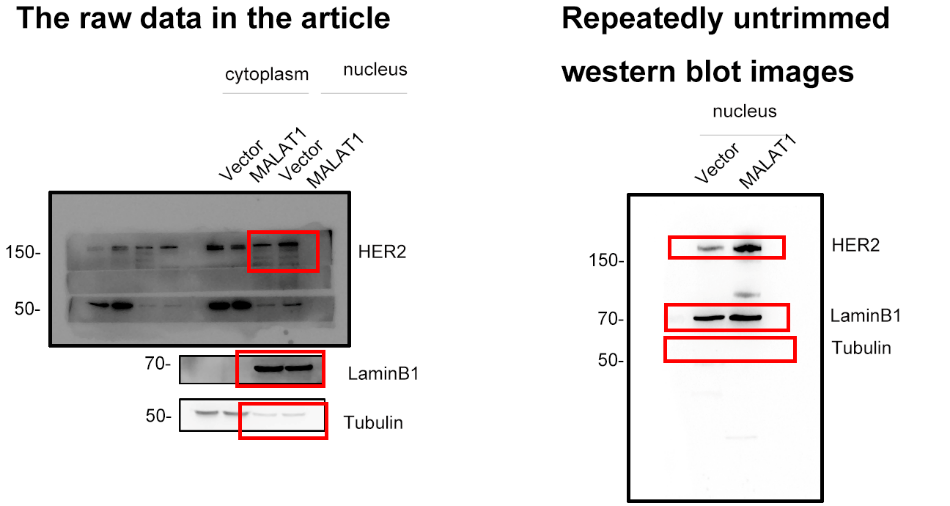
**

**2I**

**
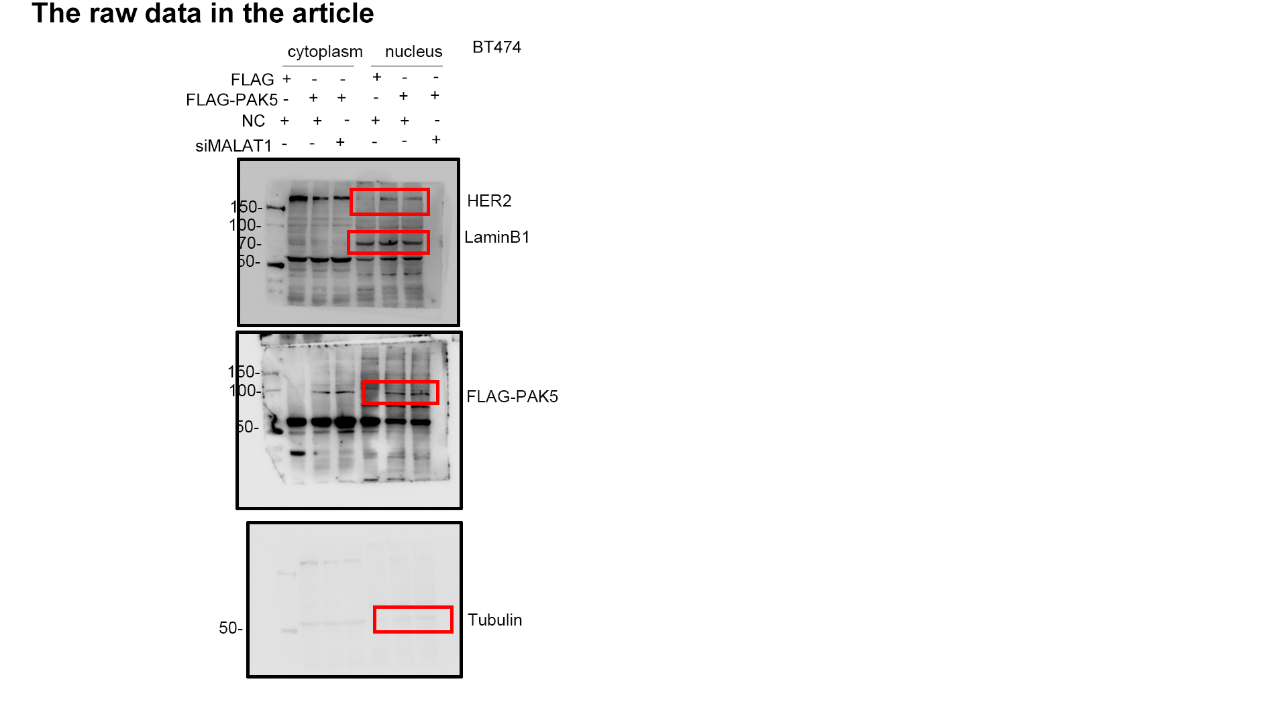
**

**3B**


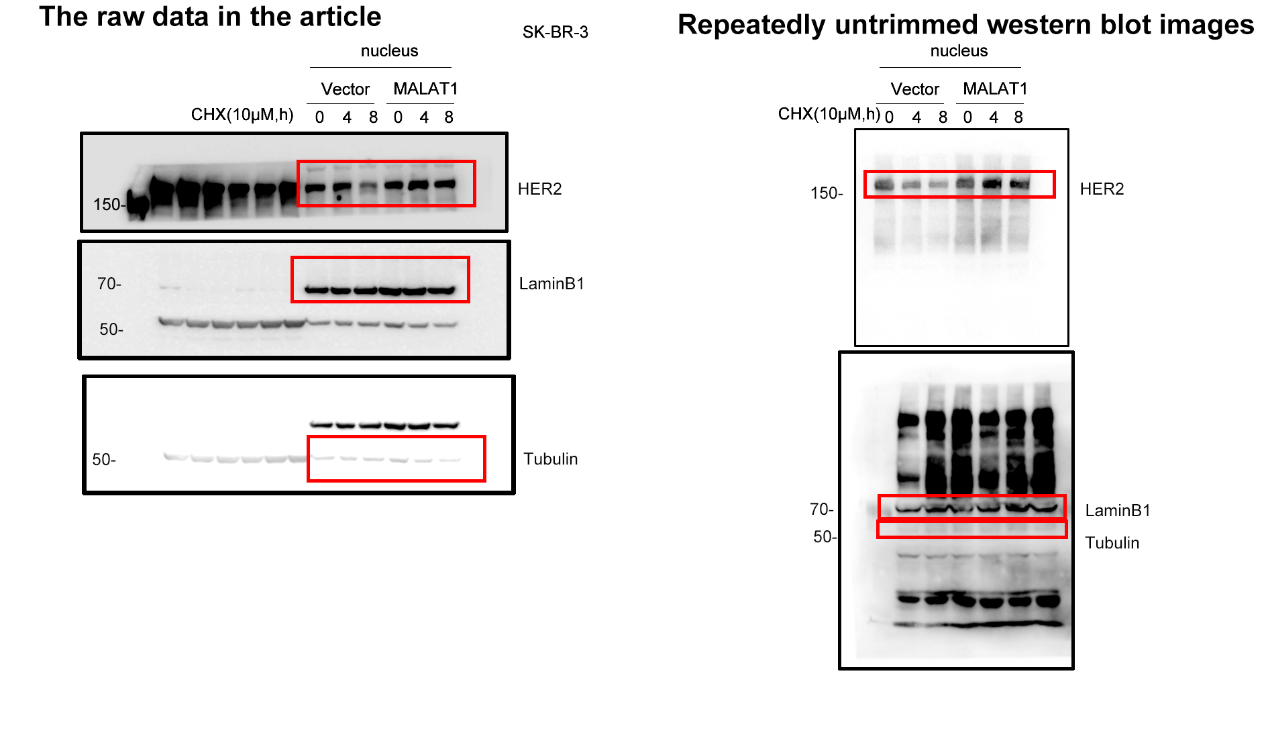


**3C**


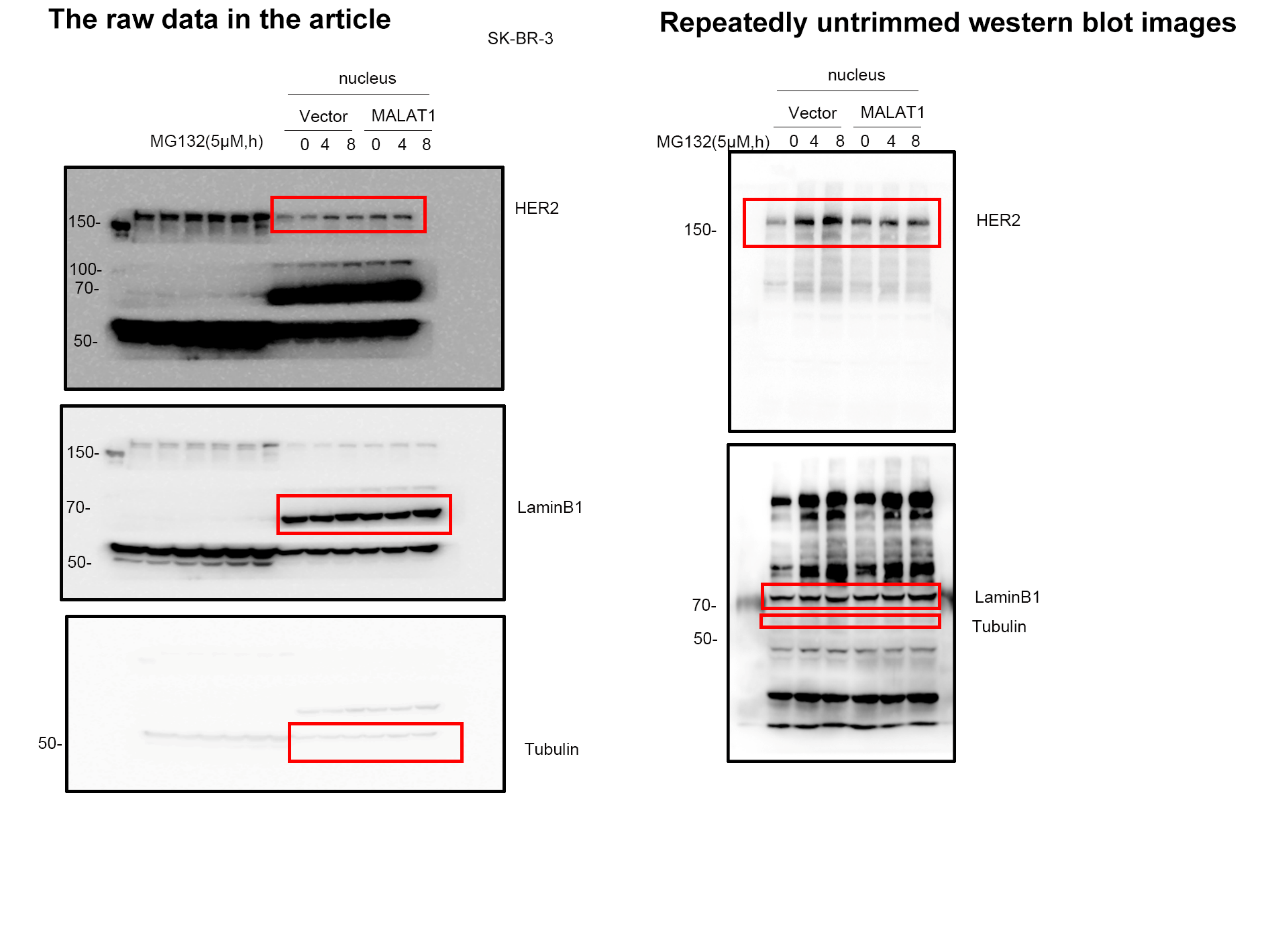


**3D**

**
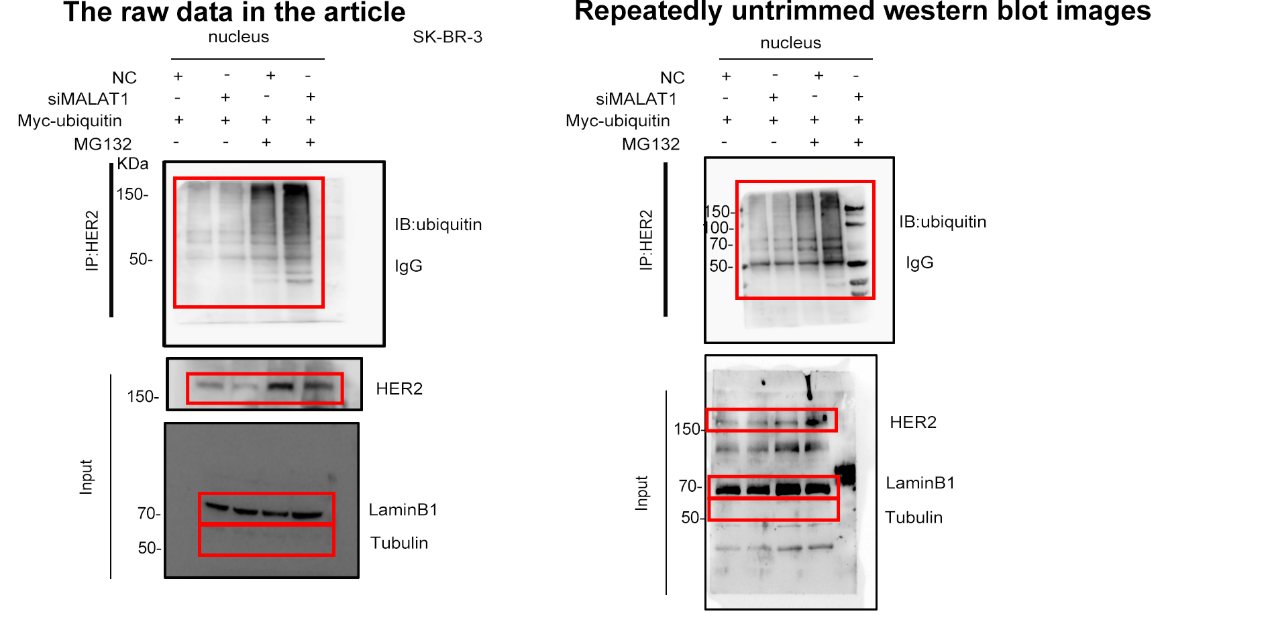
**

**3E**

**
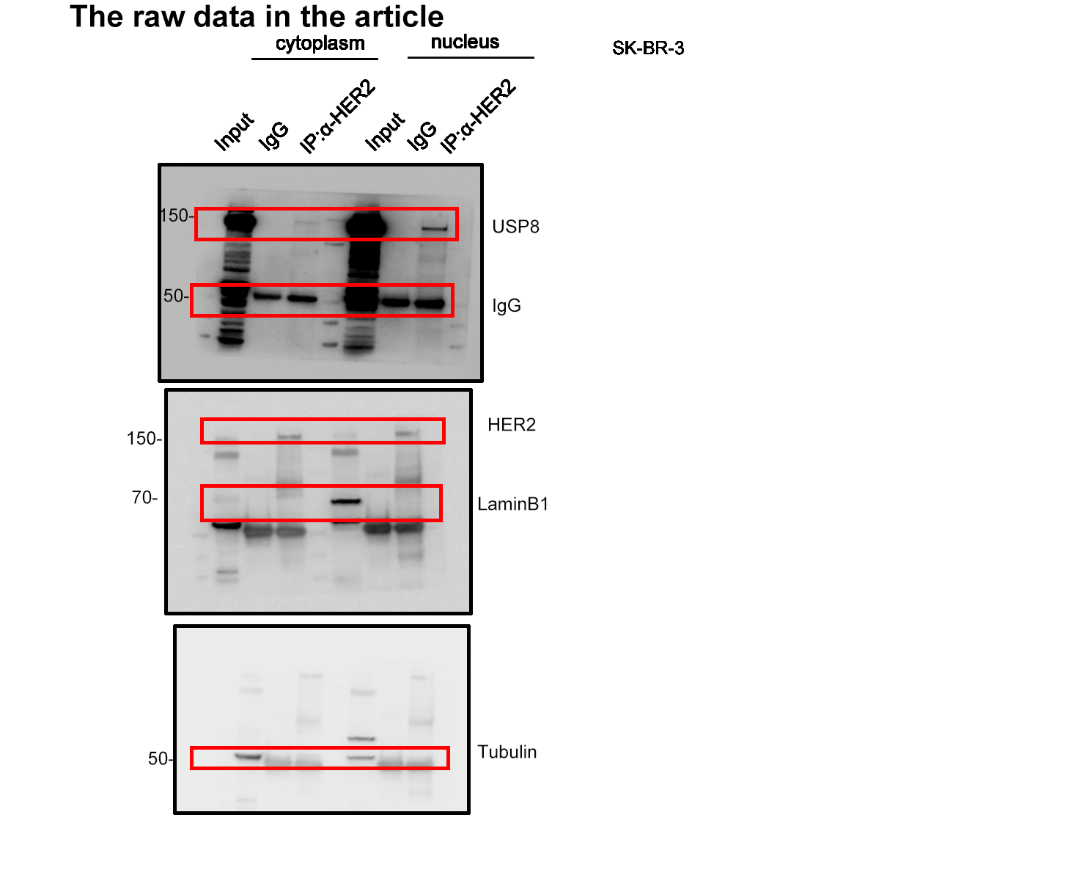
**

**3F**


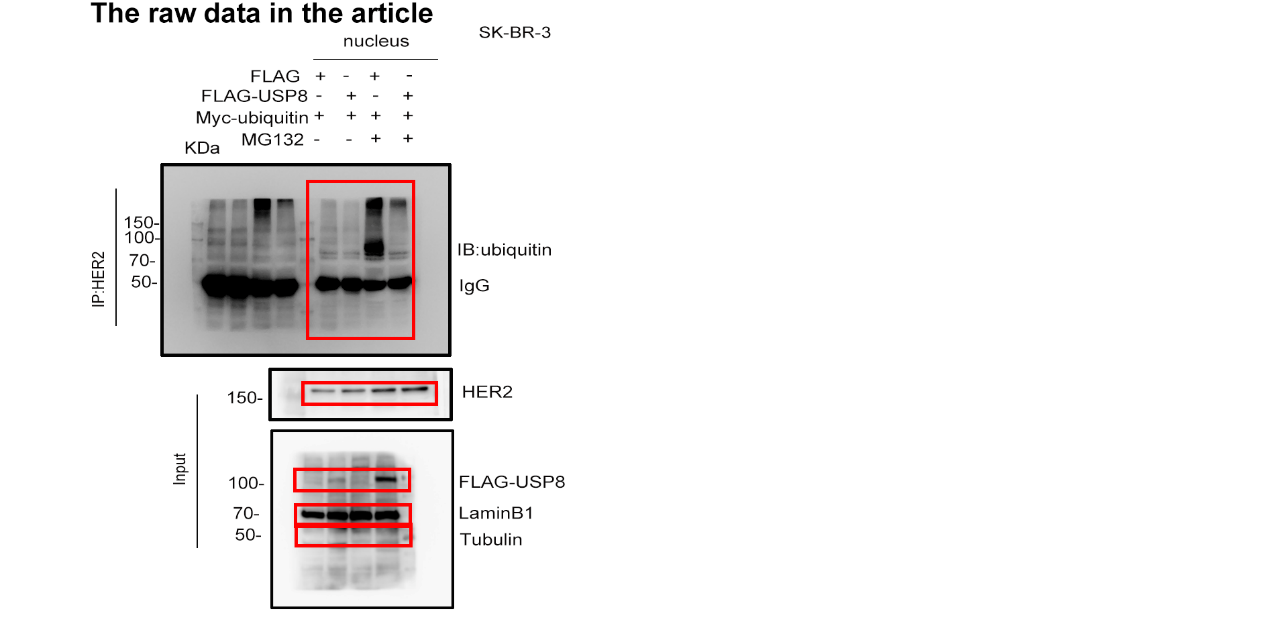


**3G**

**
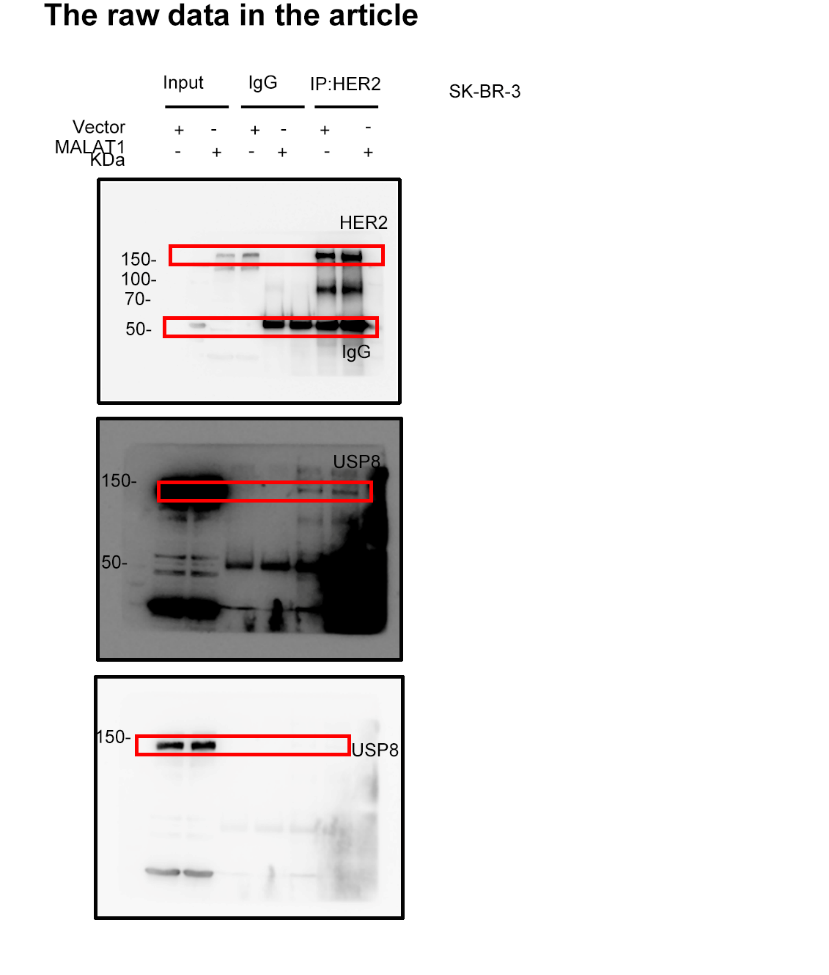
**

**3H**

**
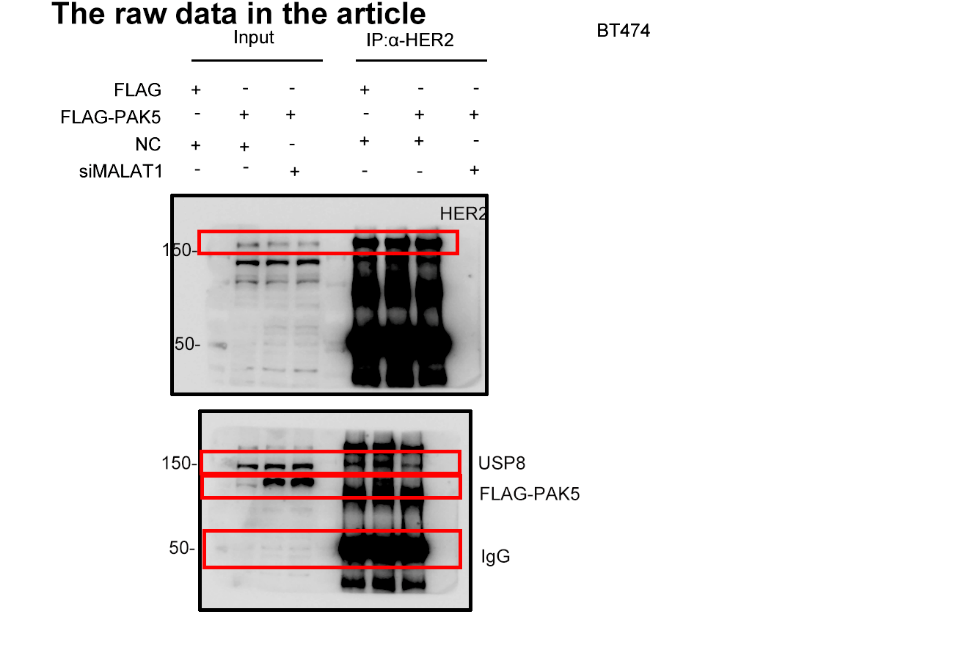
**

**4E**

**
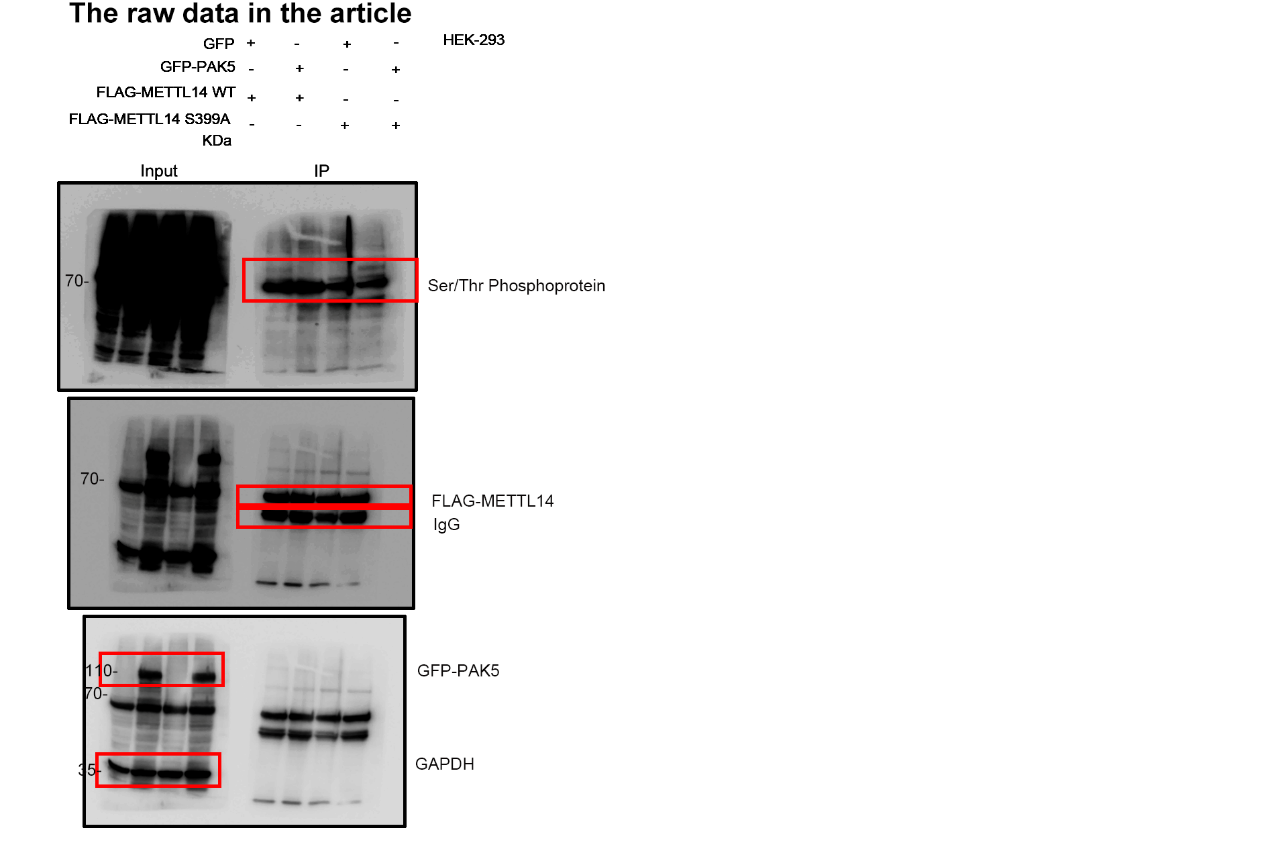
**

**6I**


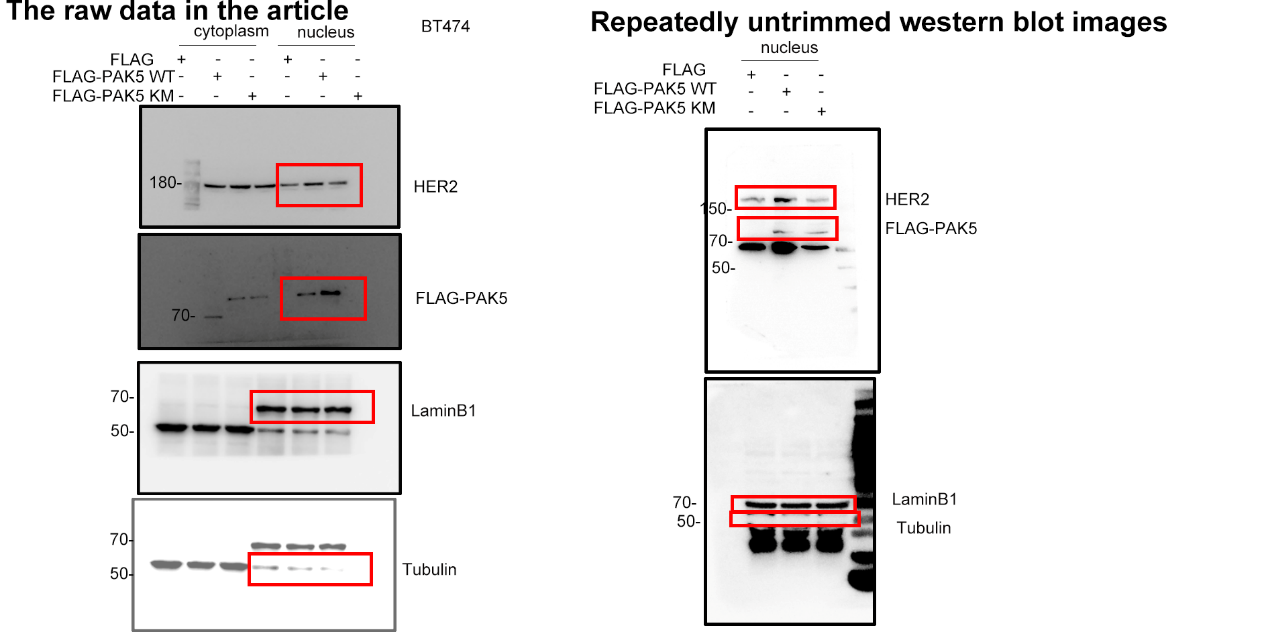


**6J**

**
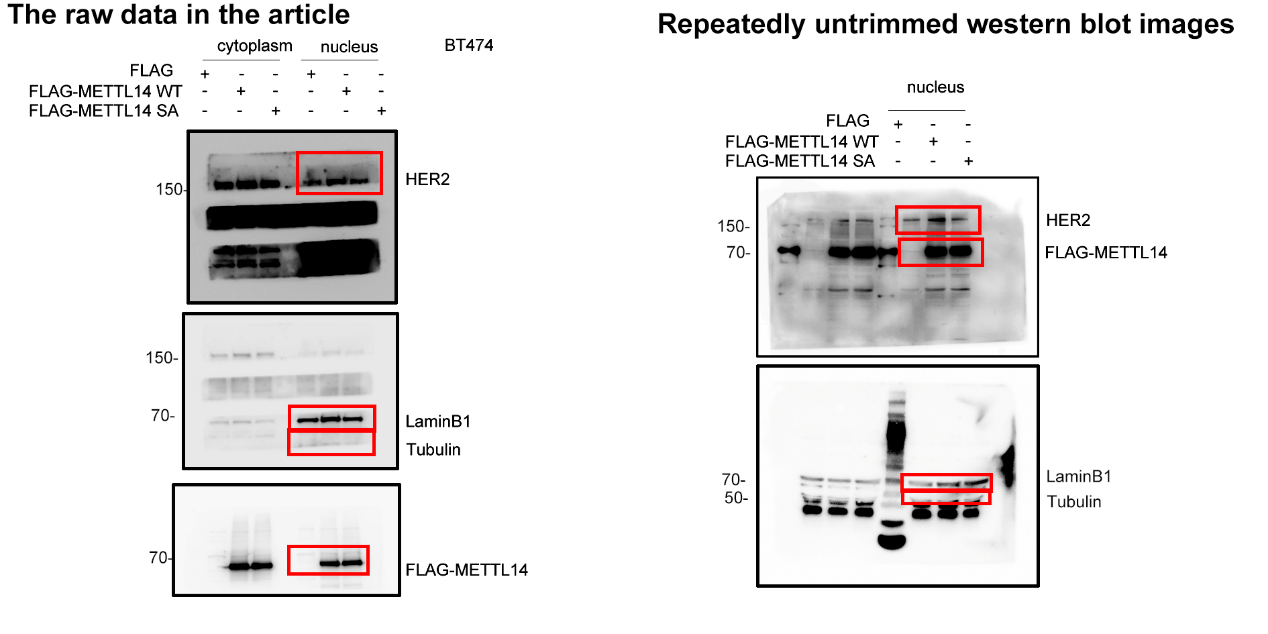
**

**6K**

**
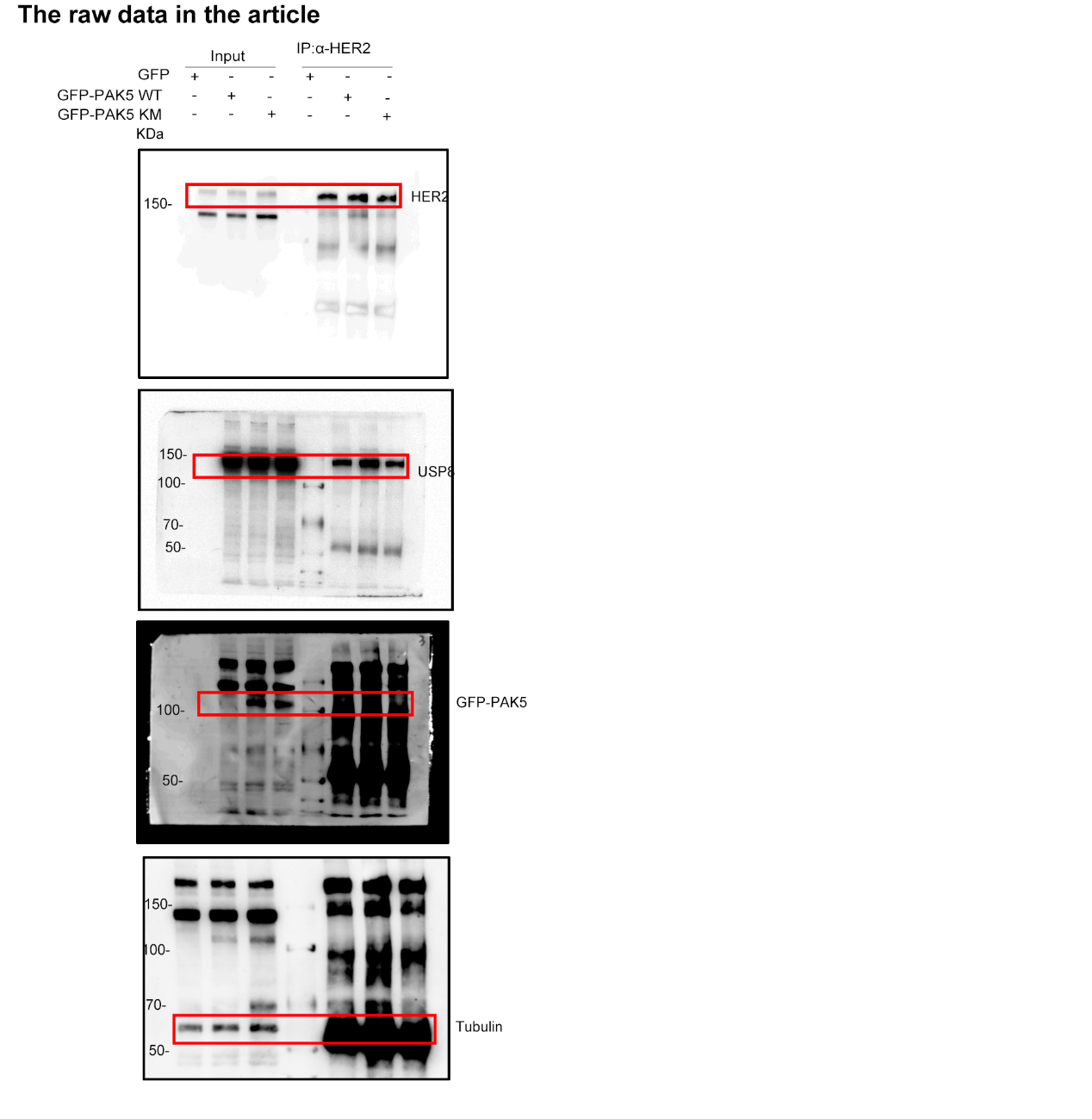
**

**7D**

**
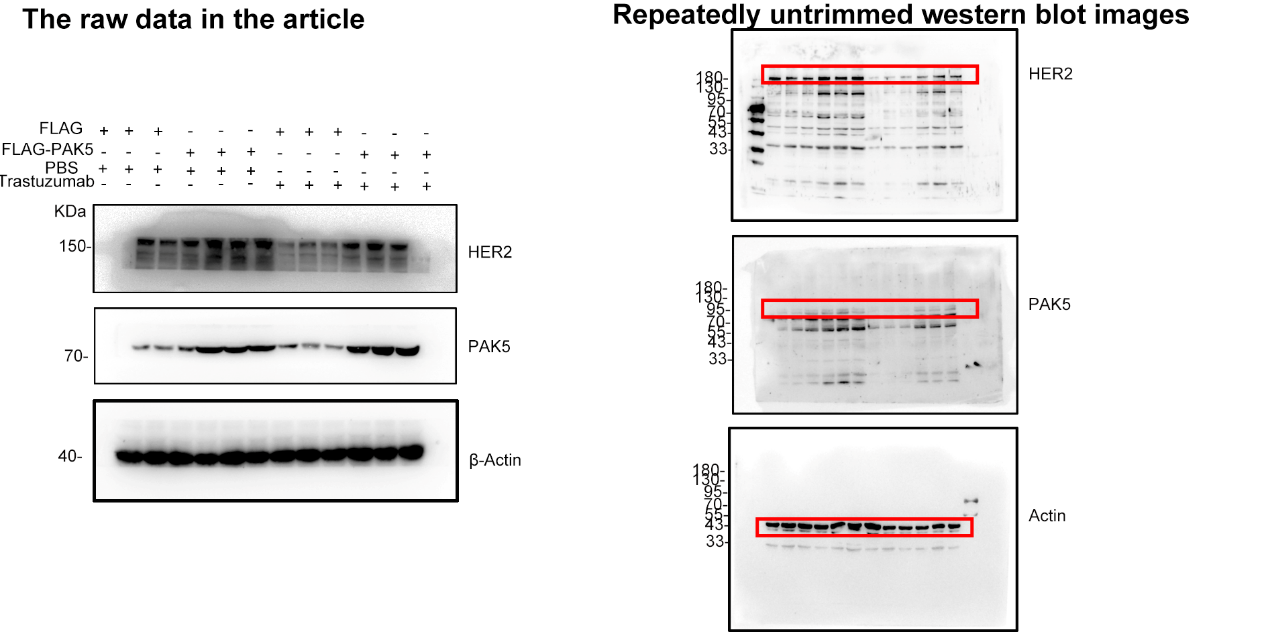
**

**7E**

**
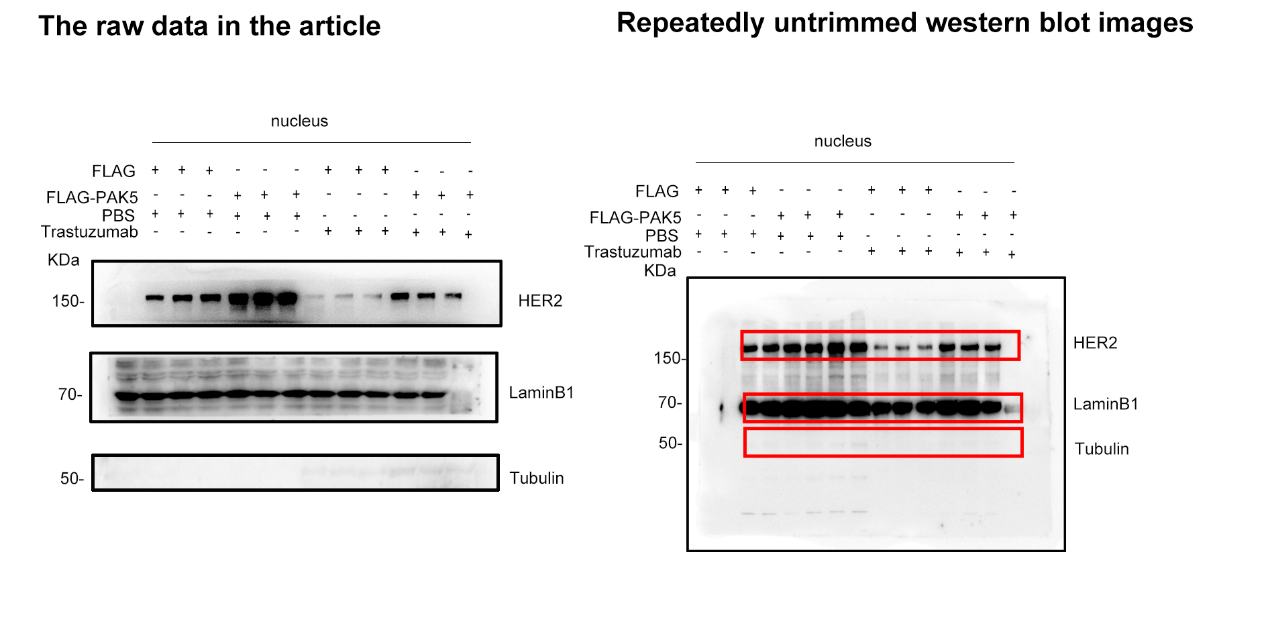
**

**7I**

**
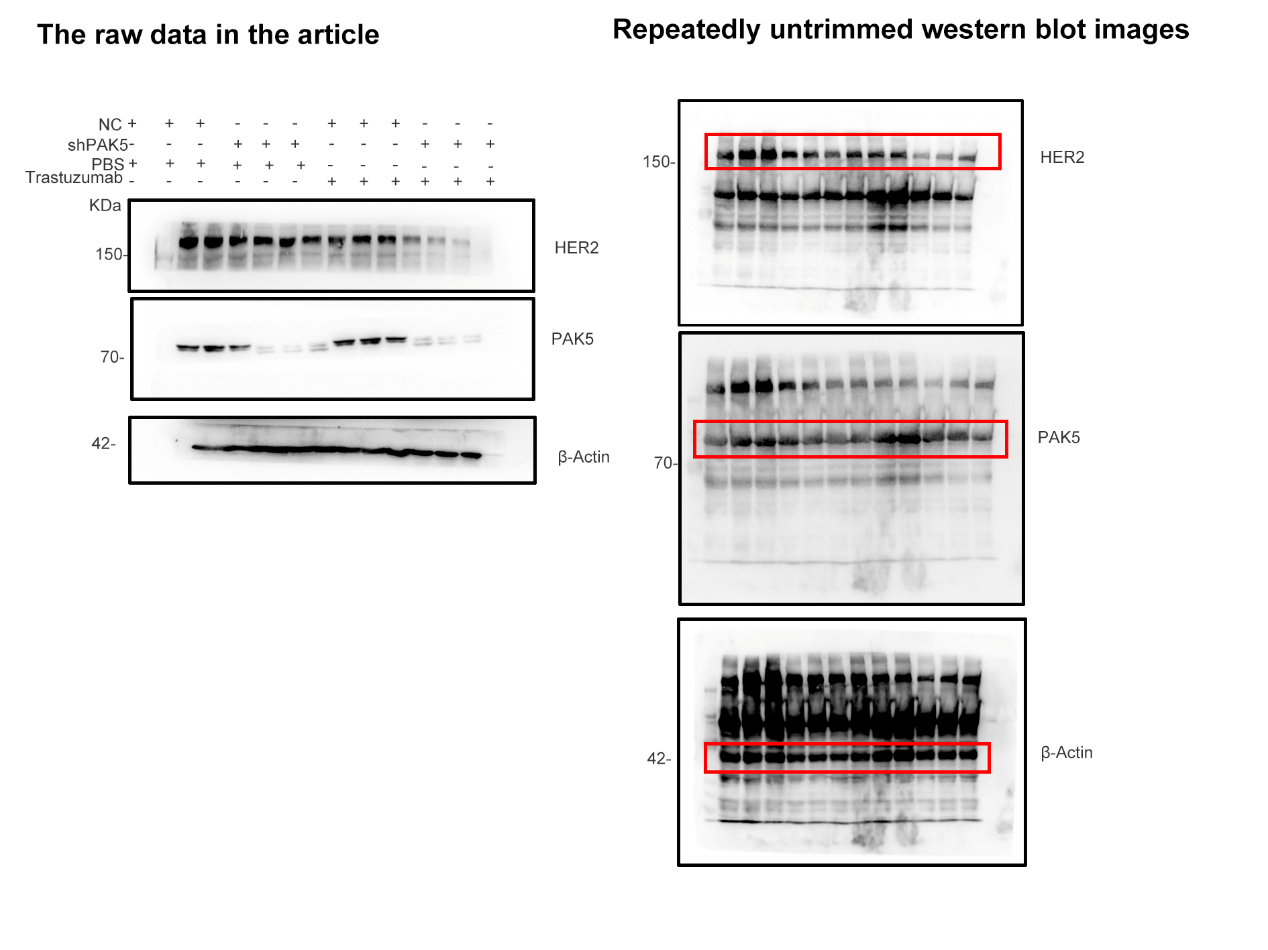
**

**7J**

**
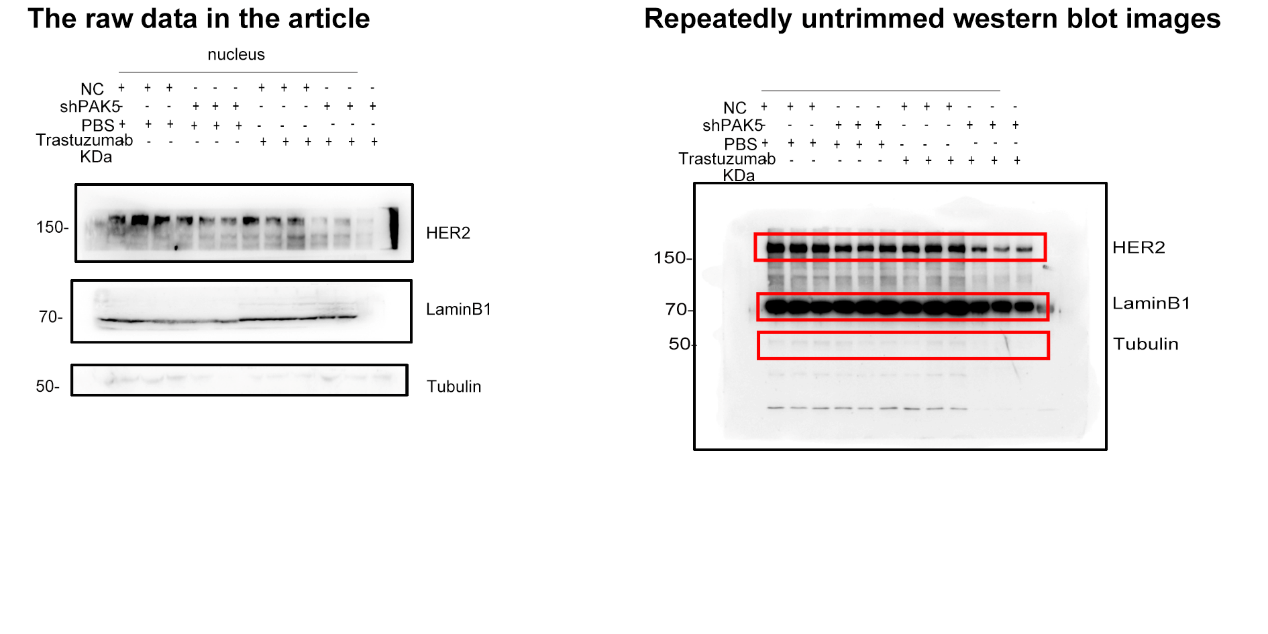
**

**S1A**

**
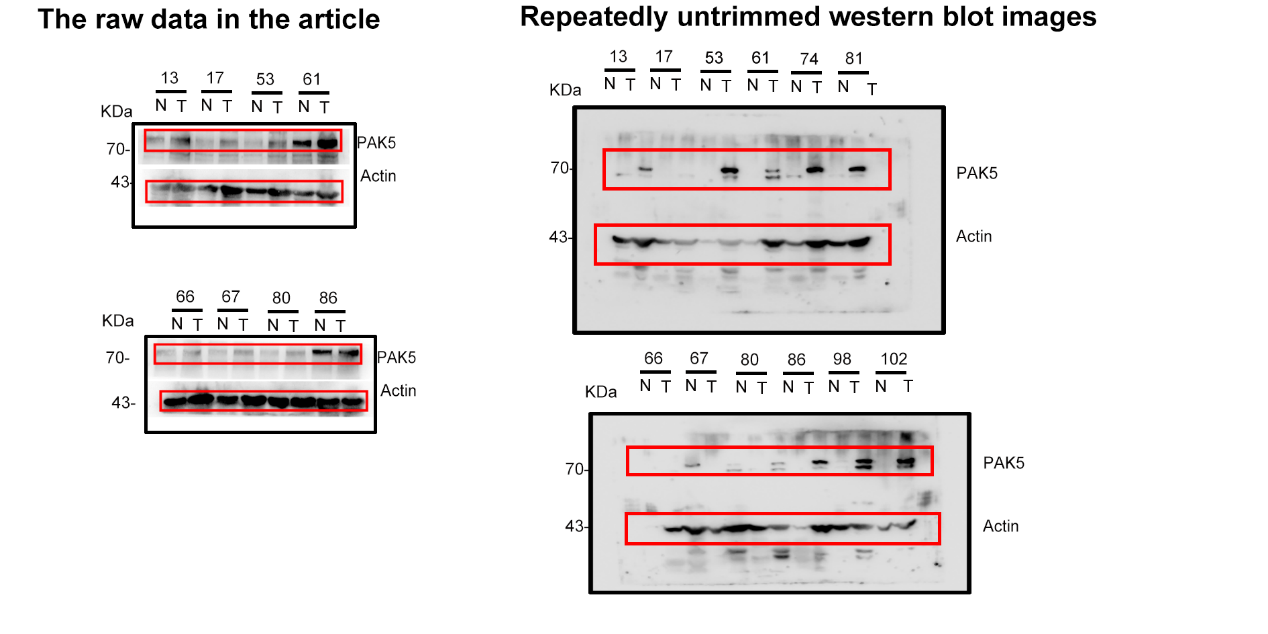
**

**S1B**

**
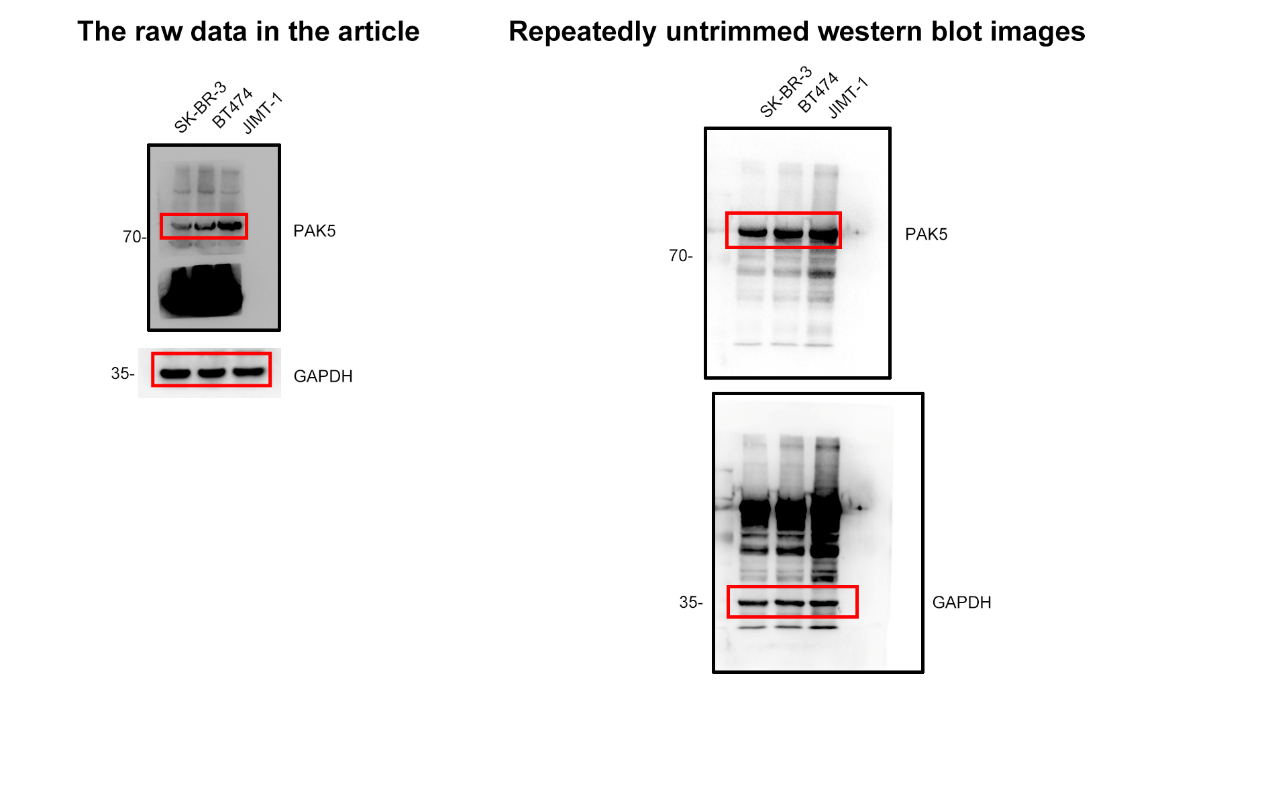
**

**S1C/S1D**

**
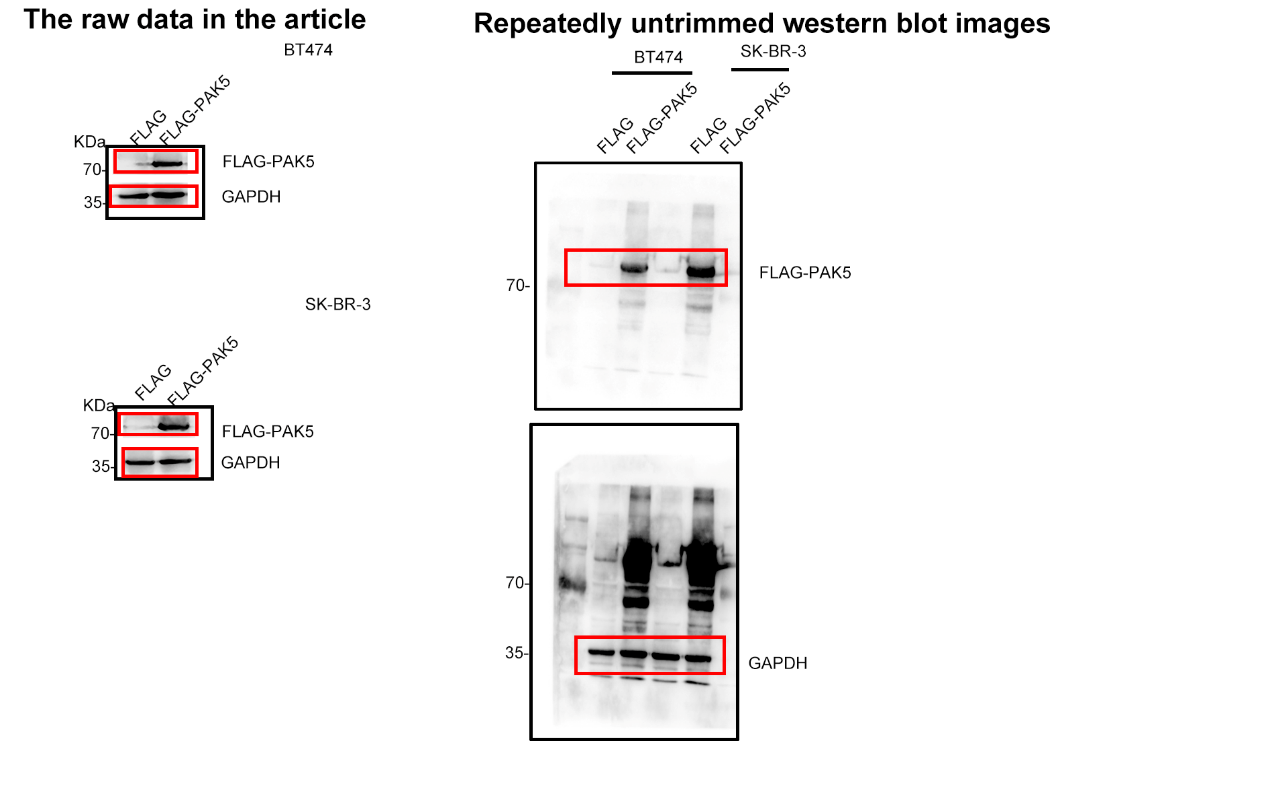
**

**S1E**

**
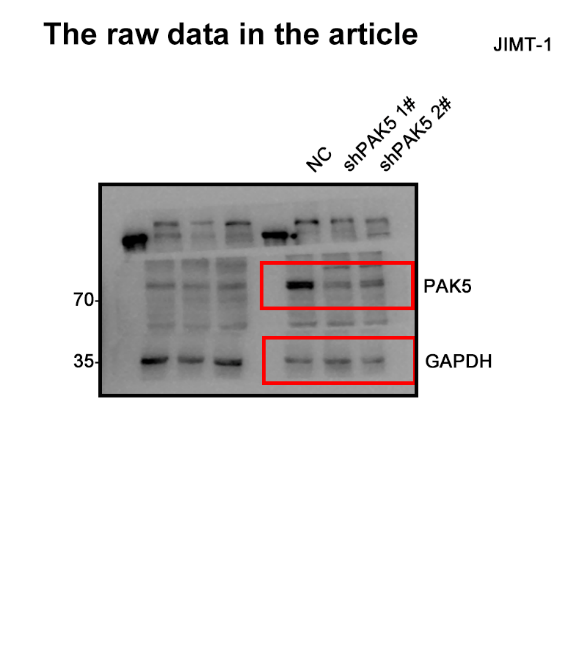
**

**S2D**

**
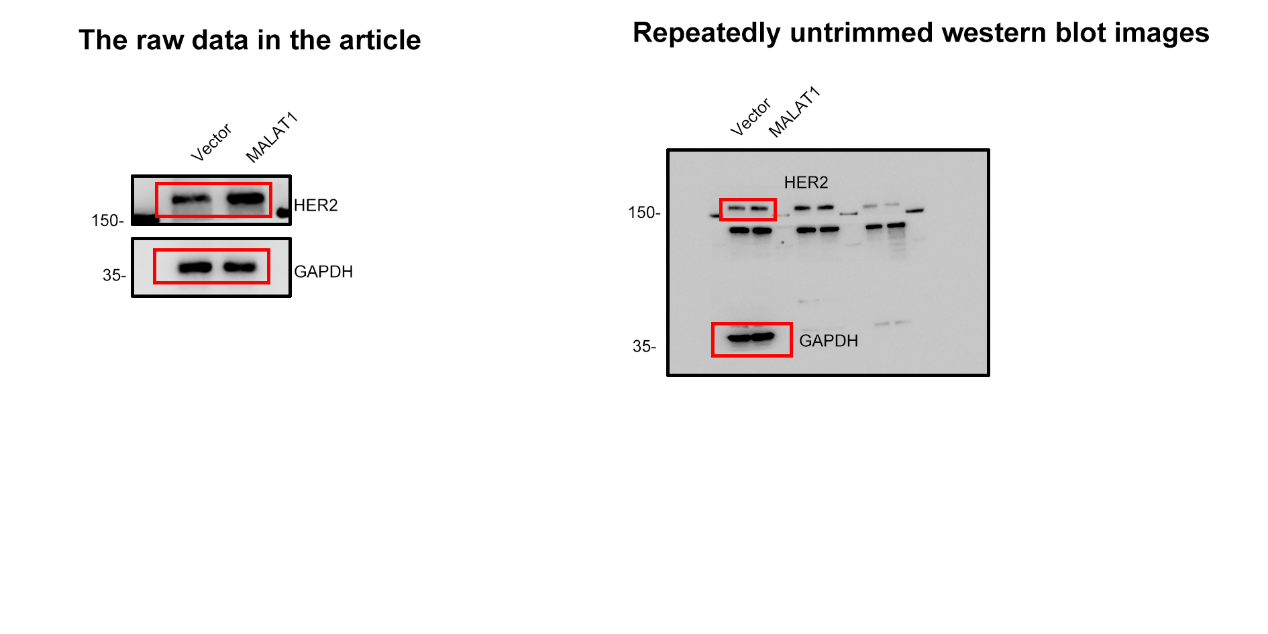
**

**S2E**


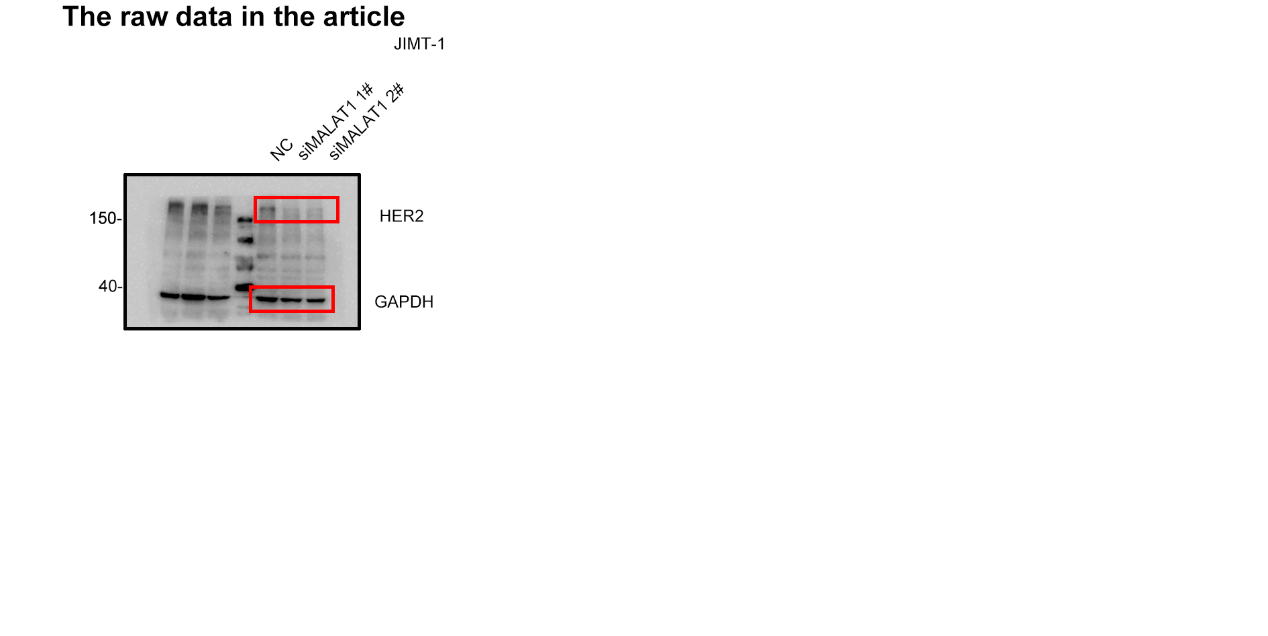


**S2J**

**
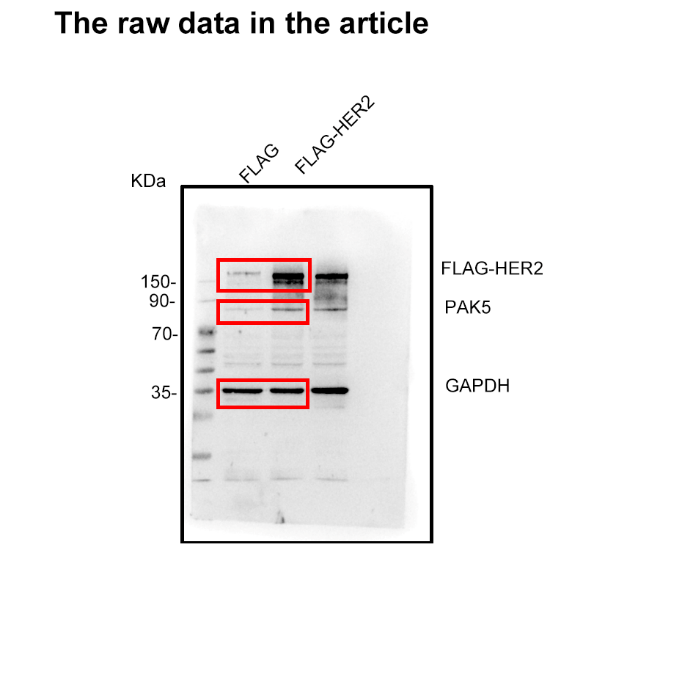
**

**S3A**

**
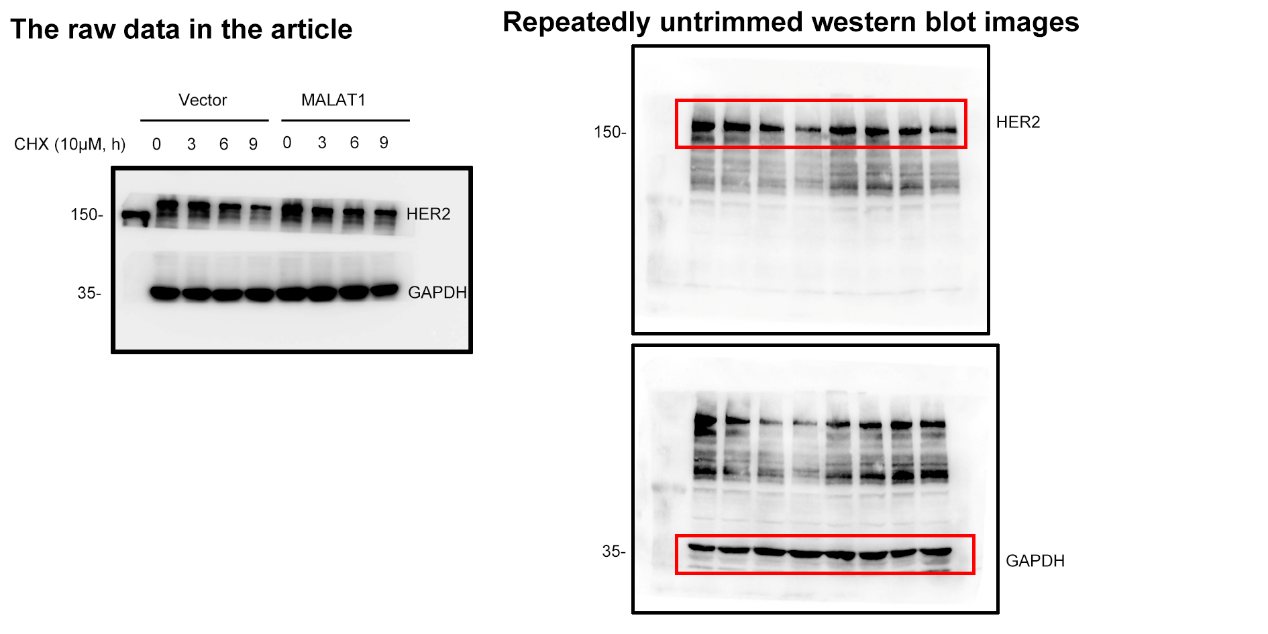
**

**S3B**

**
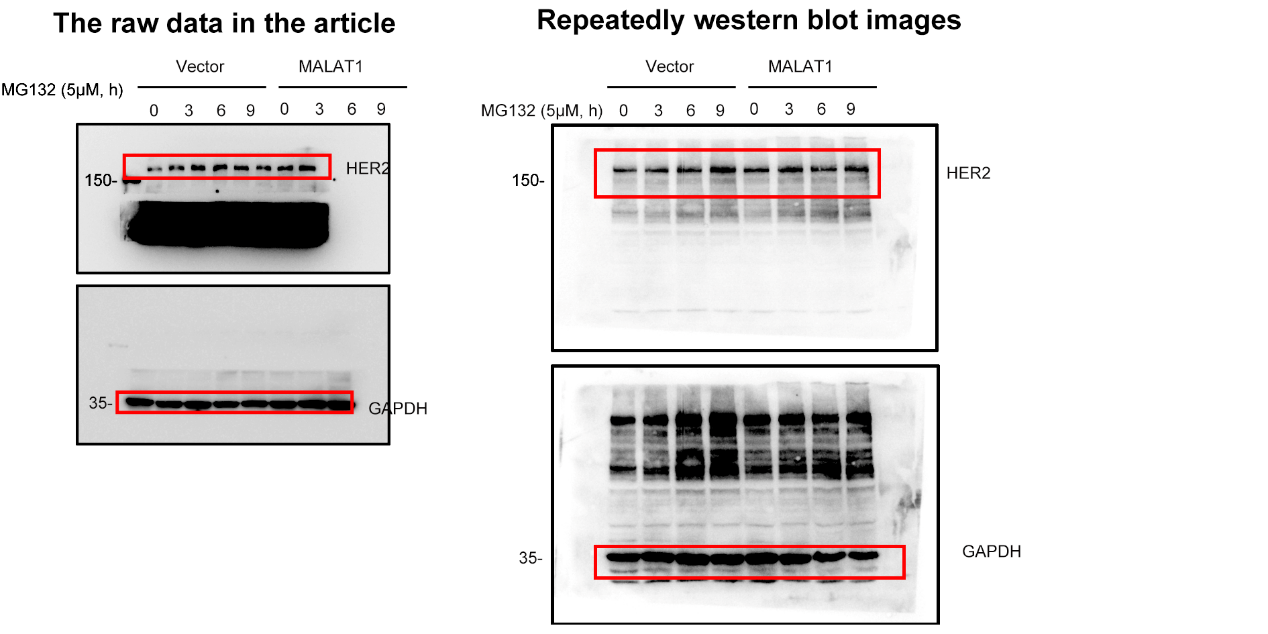
**

**S3C**

**
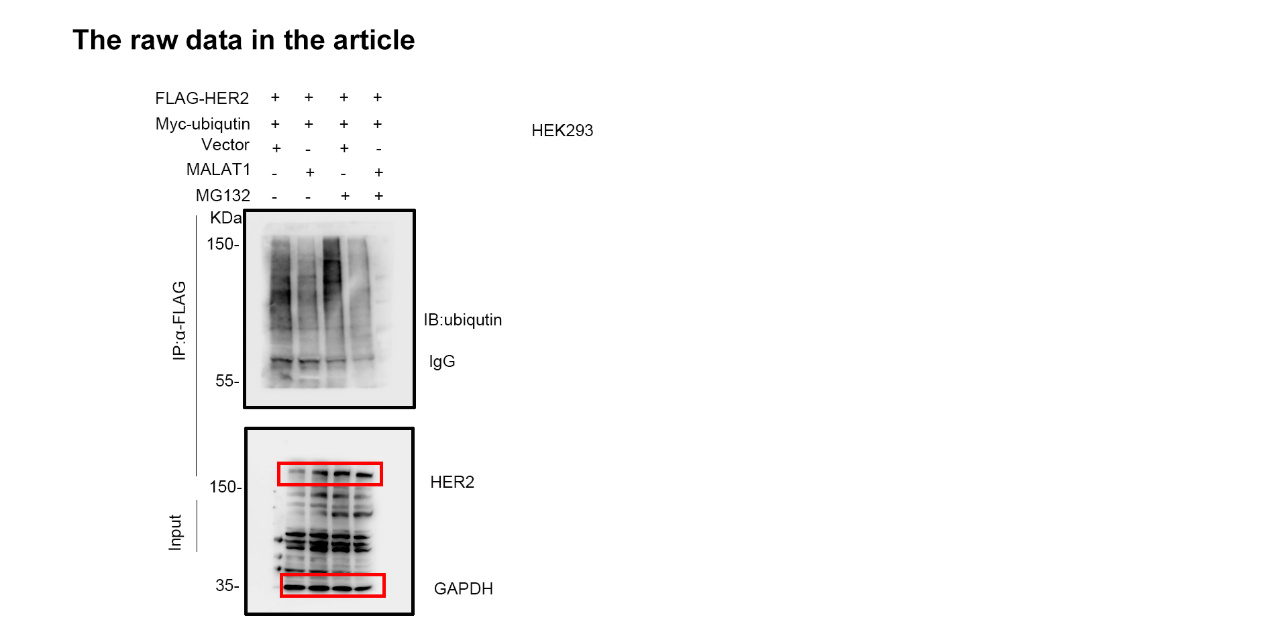
**

**S4C**

**
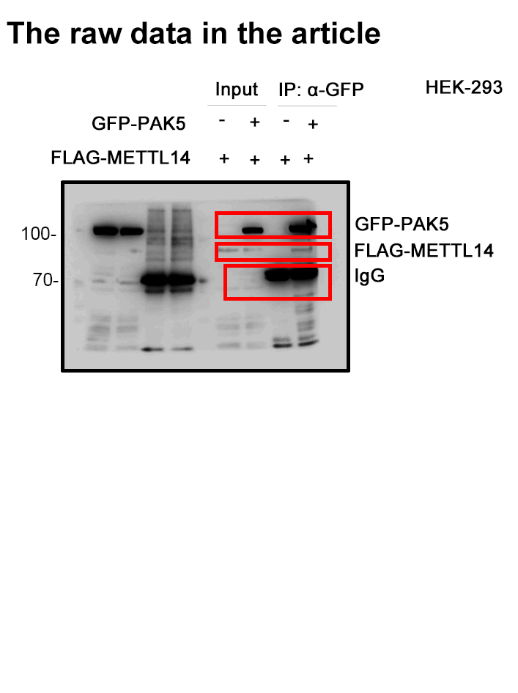
**

**S4D**

**
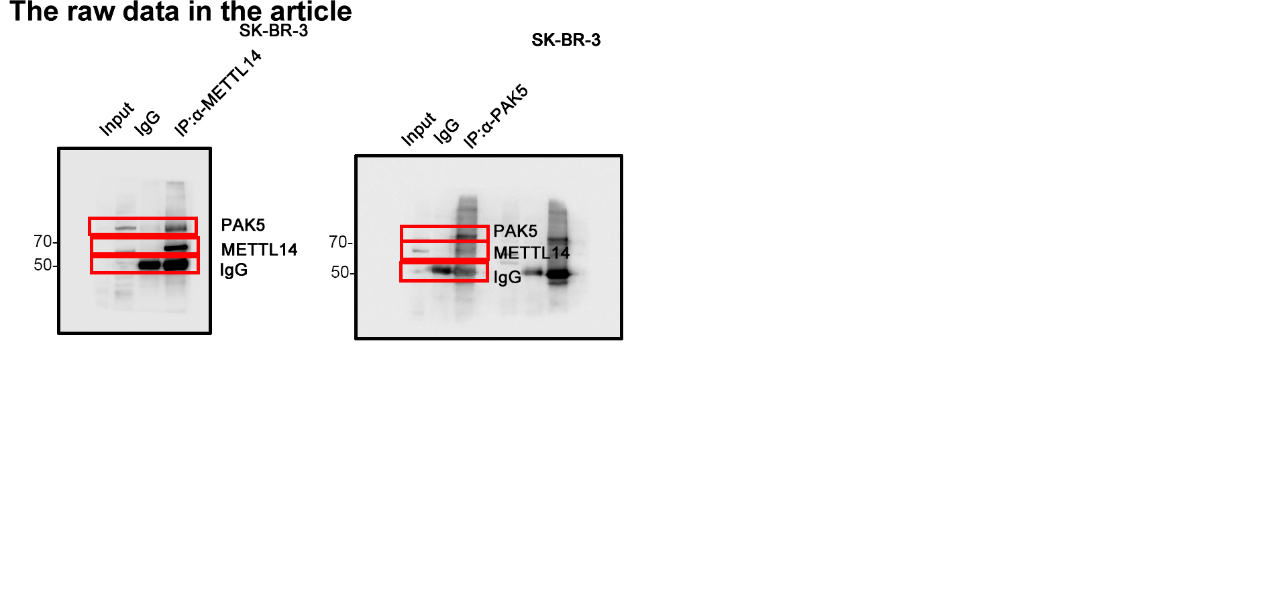
**

**S4E**

**
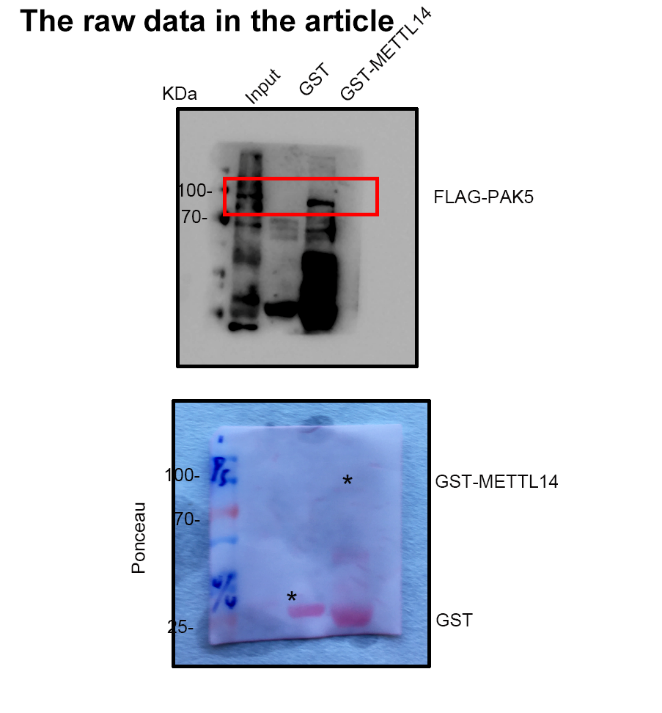
**

**S5**

**
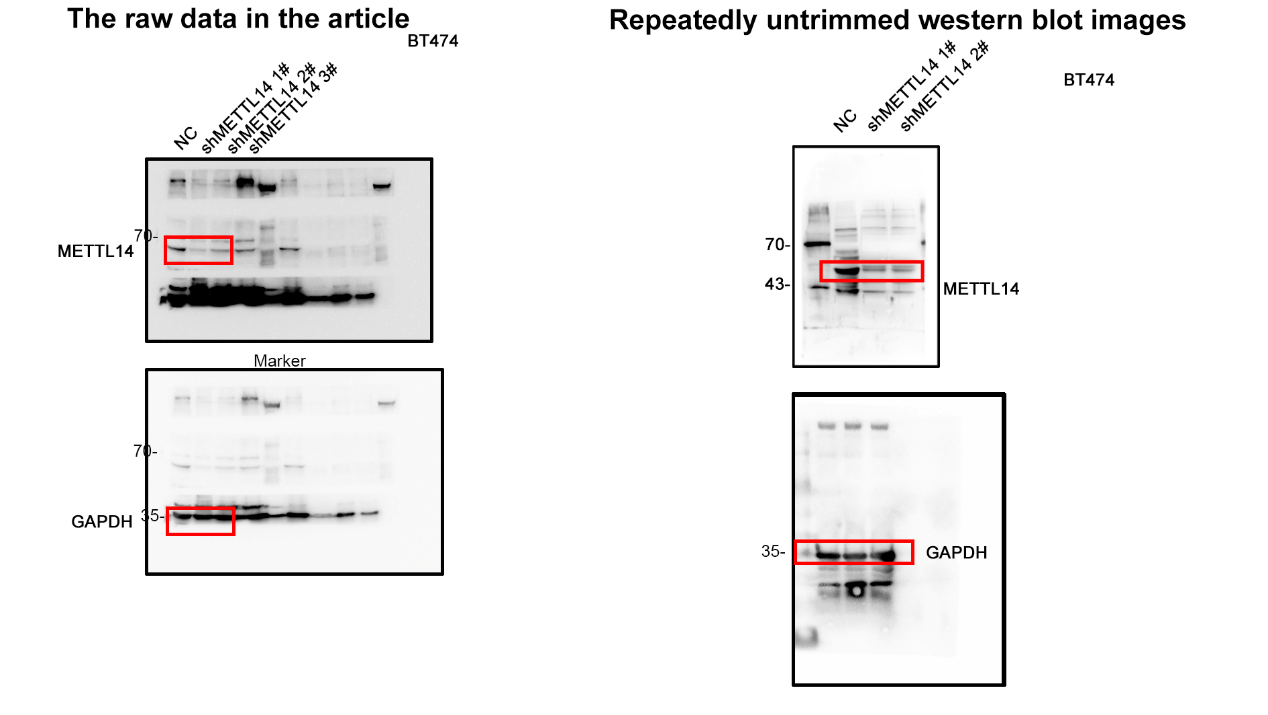
**

**S6**

**
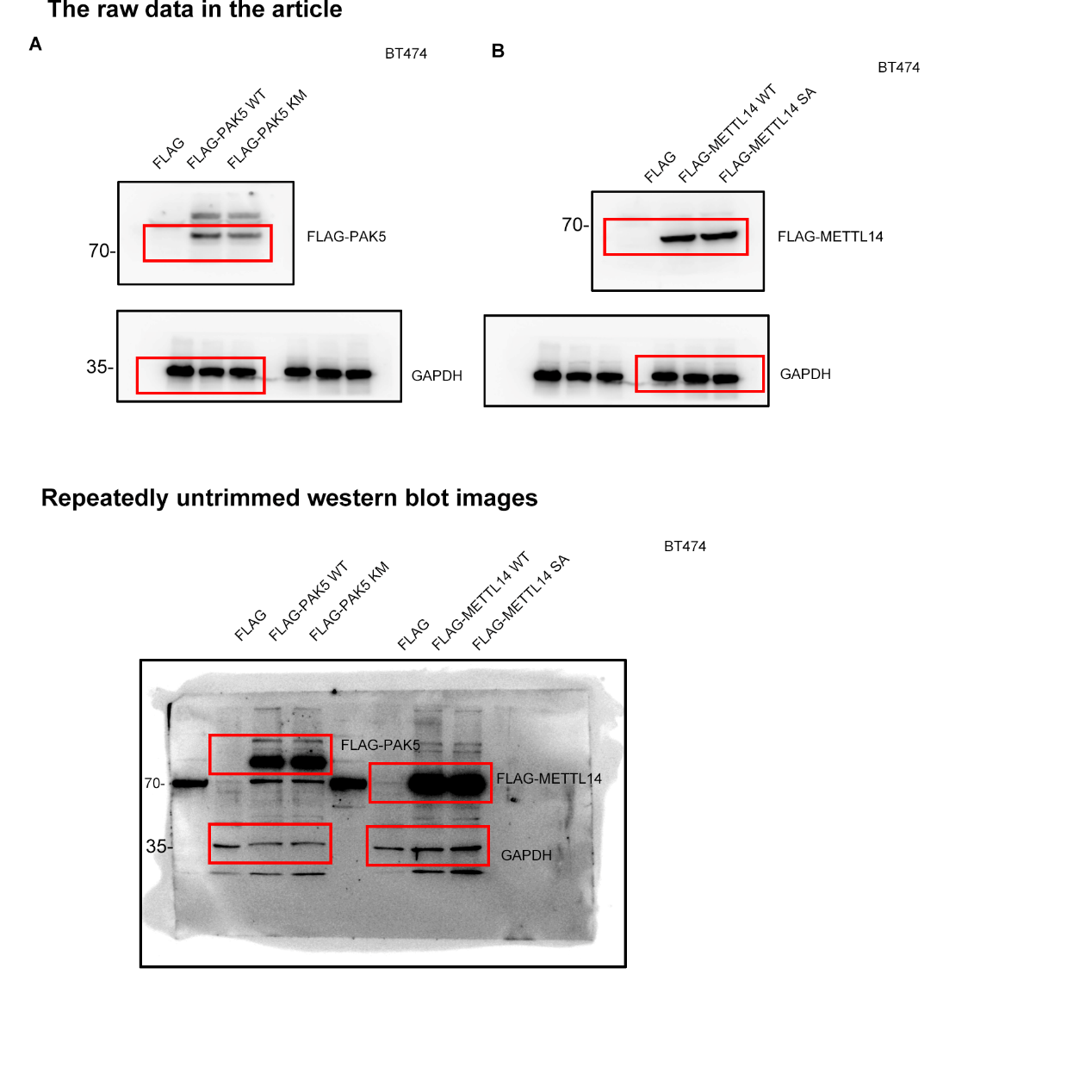
**

Supplement: Supplementary file 2 — Original western blots [file 41419_2025_7657_MOESM2_ESM.docx]
